# Large-scale comparative transcriptomic analysis of temperature-responsive genes in *Arabidopsis thaliana*

**DOI:** 10.1007/s11103-021-01223-y

**Published:** 2022-01-01

**Authors:** Napaporn Sriden, Varodom Charoensawan

**Affiliations:** 1 Doctor of Philosophy Program in Biochemistry (International Program), Faculty of Science, Mahidol University, Bangkok, 10400 Thailand; 2 Department of Biochemistry, Faculty of Science, Mahidol University, Bangkok, 10400 Thailand; 3 Integrative Computational BioScience (ICBS) Center, Mahidol University, Nakhon Pathom, 73170 Thailand; 4 Systems Biology of Diseases Research Unit (SyBID), Faculty of Science, Mahidol University, Bangkok, 10400 Thailand

**Keywords:** *Arabidopsis thaliana*, Temperature responses, Transcriptomes, Comparative omics, Heat-shock factor (HSF)

## Abstract

**Key message:**

Comparative transcriptomic analysis provides broad and detailed understandings of transcriptional responses to a wide range of temperatures in different plant tissues, and unique regulatory functions of temperature-mediating transcription factors.

**Abstract:**

Climate change poses a great threat to plant diversity and food security. It is thus of necessity to understand the molecular mechanisms for perceiving and responding to adverse temperature changes, to develop the cultivars that are resilient to these environmental stresses. Making use of publicly available datasets, we gathered and re-analyzed 259 individual transcriptomic profiles from 139 unique experiments of *Arabidopsis thaliana*’s shoot, root, and seedling tissues, subjected to a wide variety of temperature conditions, ranging from freezing, cold, low and high ambient temperatures, to heat shock. Despite the underlying differences in the overall transcriptomic profiles between the plant tissues, we were able to identify distinct sets of genes whose transcription patterns were highly responsive to different types of temperature conditions, some were common among the tissues and some were tissue-specific. Interestingly, we observed that the known temperature-responsive genes such as the heat-shock factor (HSF) family, were up-regulated not only in response to high temperatures, but some of its members were also likely involved in the cold response. By integrating the DNA-binding specificity information of the key temperature transcription factor (TF) HSFA1a, PIF4, and CBFs, we elucidated their distinct DNA-binding patterns to the target genes that showed different transcriptional responses. Taken together, we have comprehensively characterized the transcription patterns of temperature-responsive genes and provided directly testable hypotheses on the regulatory roles of key temperature TFs on the expression dynamics of their target genes.

**Supplementary Information:**

The online version contains supplementary material available at 10.1007/s11103-021-01223-y.

## Introduction

Temperature is one of the most influential environmental factors that mediate plant growth and development. Diurnal and seasonal fluctuations of surface temperature play a crucial role in determining developmental stages of plants, allowing them to grow and reproduce at suitable times (Casal and Balasubramanian 2019; Gil and Park 2019; Jenkitkonchai et al. 2021; Quint et al. 2016). As important as the natural temperature variations, drastic changes due to global warming have forced all the life forms, especially sessile organisms like plants, to either adapt to sudden fluctuations of temperature or encounter extinction, not to mention the strong detrimental effect on crop species in terms of reduced yields (Peng et al. 2004; Zhao et al. 2017).

Temperature changes from the optimal range can influence plant development and survival in several ways. High temperatures, for instance, can be largely categorized into “high ambient” temperature, typically 5–6 °C above the optimum, and “heat shock”, which is above the ambient condition (Li et al. 2018). For the model plant *Arabidopsis*, 21–22 °C are considered as the optimal condition, 27–28 °C as the typical high ambient temperature, and 28 °C or above as the heat shock temperature (Li et al. 2018). Despite only a few centigrade differences, the morphological responses under the high ambient and heat shock conditions can be very different. High ambient temperature generally promotes plant growth in several aspects, including accelerating hypocotyl elongation and flowering (Gil and Park 2019; Quint et al. 2016); whereas heat shock normally triggers stress responsive pathways, damages several cellular components, and thus hinders growth and seed production (Hasanuzzaman et al. 2013; Ohama et al. 2017).

Heat shock transcription factor A1s (HSFA1s) are transcription factors (TFs) that have been shown to play an essential regulatory role in the heat shock conditions (Liu and Charng 2012; Ohama et al. 2017; Yoshida et al. 2011). It is not yet clear; however, if the elevated expression of the heat shock factors (HSFs) and their target genes, such as the heat shock proteins (HSPs), is specific to heat shock, or is already induced under high ambient temperature. On the other hand, the transcription factor phytochrome interacting factor 4 (PIF4) is known as a central regulatory hub that integrates thermoresponsive pathways at high ambient temperatures, which also interplays with light, hormone, and circadian signaling (Casal and Balasubramanian 2019; Choi and Oh 2016; Quint et al. 2016). Earlier studies have shown that the *PIF4* gene is up-regulated in high ambient temperatures (Koini et al. 2009; Kumar et al. 2012), and mediates thermoresponsive growth through auxin biosynthesis (Franklin et al. 2011; Sun et al. 2012), but it remains unclear why PIF4 does not play the same role under heat stress conditions.

Temperatures lower than the optimum not only reduce enzymatic activities and biochemical reactions, but also adversely affect the growth and development of plants (Hasdai et al. 2006). In *Arabidopsis*, the temperature around 14 °C is considered “low ambient” or “chilling”; whereas the temperature from 6 °C down to zero is known as “cold”, which can reduce chlorophyll and anthocyanin contents, as well as delay the flowering time (Hasdai et al. 2006). More deleterious effects of low temperature were seen at “freezing” (0 °C and lower), where the damage is not only from a very low temperature, but also from ice crystalline formation that causes osmotic dehydration in plant cells (Thomashow 1999). One of the well-known regulators of the low temperature response in plants are the C-repeat binding factors or dehydration-responsive element-binding protein 1 (CBF/DREB1) TFs, which activate the transcription of multiple cold-responsive genes, including the *cold-regulated* (*COR*) genes (Stockinger et al. 1997). The transcriptional levels of *CBFs/DREB1s* can be induced at low ambient (17 °C) and cold temperatures (4 °C) (Dong et al. 2020; Novillo et al. 2007). Despite some prior knowledge about their functions, it remains to be seen how the expression of *CBFs* and their target genes is altered at different ranges of low temperature conditions.

Thanks to the wealth of publicly available high-throughput omic data, we now have an unprecedented opportunity to combine and investigate multiple gene expression datasets, and characterize the common and unique pathways that organisms employ to perceive and respond to different stresses. In plants, comparative genomics and transcriptomics have been utilized to a great effect to elucidate the dynamic expression in response to external stimuli, such as abiotic stresses (Sharma et al. 2018; Shen et al. 2017; Yadav et al. 2016), and pathogenic infection (Jiang et al. 2017). However, to the best of our knowledge, such comprehensive analyses of multiple transcriptomes of plants grown under different temperature conditions are yet to be reported, despite a large body of temperature transcriptomic data publicly available.

Taking advantage of the existing temperature transcriptomes, from both expression microarray and RNA sequencing (RNA-seq), we have gathered, re-normalized, and unbiasedly re-analyzed the integrated transcriptomic profiles of *Arabidopsis thaliana* subjected to a wide range of temperature conditions and treatments, ranging from freezing, cold, low ambient, high ambient, and heat shock temperatures. Despite the overall transcriptomic patterns largely grouped by the plant tissues, we were able to characterize clusters of genes with distinct transcription patterns associated with different temperature profiles and treatments. In addition to the comparative transcriptomic analyses, we could link the DNA-binding profiles of key TFs known to be important to the temperature-responsive pathways of plants, namely HSFA1a, PIF4 and CBFs, to the temperature-specific gene clusters identified by their unique transcriptomic patterns. Taken together, we have comprehensively integrated and analyzed the transcriptomic datasets of the model plant *Arabidopsis* subjected to multiple temperature conditions, and demonstrated how the integrated transcriptomic profiles can be used to explore new transcriptionally and functionally related genes and pathways across the stress conditions of interest.

## Materials and methods

### Transcriptomic data pre-processing and analyses

A summary of all the analyses and datasets can be found in Fig. S1. The publicly available transcriptomic data (microarray and RNA-seq) used in this study were also described in Table S1. The microarray datasets in the .CEL format of the *A. thaliana* ecotype *Col-0*, grown under different temperature conditions or subjected to different temperature treatments, were obtained from Gene Expression Omnibus (GEO, see Table S1 for the summary). There were 153 individual arrays in total (including biological replicates) from 66 unique experimental set-ups. All the datasets were analyzed using the ATH1-121501 Affymetrix Arabidopsis ATH1 Genome Array (GEO microarray platform: GPL198), which contains 22,810 probes and 21,314 unique genes. The raw data were simultaneously normalized using the robust multi-array average (RMA) method (Irizarry et al. 2003) to account for the biases between different batches and experiments. The RMA-normalized transcription values of the genes that were represented by more than one probe were collapsed into a single value using the mean value, resulting in 21,314 unique genes in total. For the RNA-seq data, the raw reads in the fastq format were downloaded from the Sequence Read Archive (SRA), and the integrated dataset comprises 106 individual files from 73 unique experiments (see Table S1). Please see the details of RNA-seq data analysis in Supplementary Results.

### Identification of highly variable genes (HVGs) across the temperature profiles

To identify the genes which their transcription levels are influenced by different temperature conditions or treatments, we first extracted the “highly variable genes”, or “HVGs” herein, based on the high variations across the temperature conditions of the RMA-normalized expression values (for microarray) or the DESeq2-normalized reads (for RNA-seq). We employed two measures to assess the variations of the gene expression across multiple temperature transcriptomic datasets: standard deviations (SDs) and p-values from the ANalysis Of VAriance (ANOVA) test. HVGs were defined by their relatively high SDs and low p-values of the ANOVA tests, as compared to other genes in the genome. Due to different natures and ranges of the normalized transcription levels from different datasets, tissues of origin and transcriptomic technologies, the SD and p-value cut-offs were identified heuristically for different datasets, using the inflexion points or the “elbows” of the scree plots (Cattell 1996) (see Fig. S2). The cut-offs for HVGs were: − log p-value > 35 and SD > 1 for the shoot microarray dataset; − log p-value > 10 and SD > 1 for the root microarray dataset (see Fig. S2 for inflexion points). The RMA-normalized transcription levels from the microarray and the normalized read counts from the RNA-seq transcriptomes of the HVGs were re-normalized using z-scores to demonstrate relative up- and down-transcriptional levels, as compared to the means across all temperature conditions. These will be referred to as “normalized transcription levels”.

The normalized transcription levels of temperature HVGs were represented in heatmaps generated using the ComplexHeatmap package (Gu et al. 2016), which was implemented in R (R core team 2019). The genes with similar transcriptional patterns were hierarchically clustered using the Ward's method. To statistically test whether the temperature conditions have significant influences on the average transcription levels of a particular gene family, we performed additional ANOVA and subsequent post-hoc tests using Tukey’s HSD, Wilcoxon test, or as indicated otherwise. The complete statistical tests of the differences in transcription levels between temperature conditions of interest can be found in Table S2.

### Gene ontology (GO) enrichment analysis

A set of HVGs were individually tested for the GO term enrichment using the Singular Enrichment Analysis (SEA), available from the AgriGO v2 webtool (Tian et al. 2017). For the microarray HVGs, all the genes presented in the ATH1-121501 Affymetrix *Arabidopsis* gene chip in the TAIR genomic locus (TAIR10_2017) format were used as the reference background. Statistical testing on the GO term enrichment was performed using Fisher's exact test, with Hochberg (FDR) multi-test adjustment, and with the significant p-value cut-off of 0.05. Redundant GO terms were summarized using REViGO (Supek et al. 2011). The non-redundant GO terms and enrichment scores were visualized using the Treemap package (Tennekes and Jonge 2011).

### Analyses of DNA binding and occupancy of temperature-responsive transcription factors

Two types of DNA-binding profiles were analyzed in this study: Chromatin immunoprecipitation sequencing (ChIP-seq) of HSFA1a (Cortijo et al. 2017) and PIF4 (Oh et al. 2012); and DNA affinity purification sequencing (DAP-seq) of CBF1, CBF2 and CBF3 (O’Malley et al. 2016) (Table S3). After the quality control step using FastQC, cleaned raw reads were mapped to the TAIR10 *Arabidopsis* genome using Bowtie2 (Langmead and Salzberg 2012). The reads mapped to each base pair along the genome were counted using the genomecov function of BEDtools (Quinlan 2014), and normalized by the genome-wide average counts using our in-house Perl scripts (as in Cortijo et al. 2017). Enrichments of ChIP-seq or DAP-seq peaks were analyzed by MACS2 (Zhang et al. 2008), and the putative target genes of the peaks were identified using the ChIPseeker package (Yu et al. 2015). The average occupancy profiles of each TF for different temperature-specific gene clusters were obtained by averaging per-base ChIP/DAP signals assigned to the promoters and their proximal regions (± 500 bp from transcription start site, TSS) of the target genes using the IRanges package in R (Lawrence et al. 2013). Only the genes whose ± 500 bp regions do not overlap with the neighboring genes were used to plot the average ChIP/DAP occupancy profiles.

## Results

### Exploring the genes with variable transcription levels under various temperature conditions

We exhaustively combined and re-analyzed publicly available temperature transcriptomic profiles from microarray and RNA-seq, to unbiasedly explore and characterize temperature-responsive genes among various temperature conditions. For the microarray, we were able to gather 153 transcriptomic profiles (including biological replicates), obtained from 66 unique experimental conditions, across 14 different studies (see Table S1 for the complete list of datasets and Fig. S1 for the summary of datasets and analyses). The microarray transcriptomes can be broadly categorized into seven temperature-specific experimental conditions: freezing (< 0 °C), cold (3–4 °C), low ambient (15–17 °C), normal (20–23 °C), high ambient (25–27 °C), heat shock (> 37 °C), and heat shock followed by recovery (> 37 °C, then back to the normal temperature for 1 to 24 h).

For RNA-seq, we gathered 106 datasets from 73 unique experimental conditions, across seven different studies, which can be categorized into three broad temperature-specific conditions: low ambient (15–17 °C), normal (20–23 °C), and high ambient temperature (25–27 °C). Due to the technical and fundamental differences between the microarray and RNA-seq studies, in terms of dynamic range, ability to detect low transcript, and pre-processing methods (Zhao et al. 2014), we analyzed the transcriptomes obtained from the two platforms separately (Fig. S2). As the integrated microarray transcriptomes consist of a larger number of experiments and more diverse temperature profiles, here we focused on the microarray results, whereas those of the RNA-seq datasets can be found as Supplementary Results. The integrated transcriptomic datasets from both microarray and RNA-seq were mainly separated by the tissue types (Fig. S3). Hence, we analyzed the temperature transcriptomic patterns separately for the “shoot” (the M-S clusters) and “root” (M-R clusters) for the microarray datasets, and “seedlings” (R-S clusters) and “root” for the RNA-seq experiments (R-R clusters).

### Integrated temperature transcriptomic profiles from microarray reveal unique transcription patterns of temperature-specific clusters

**Fig. 1:**
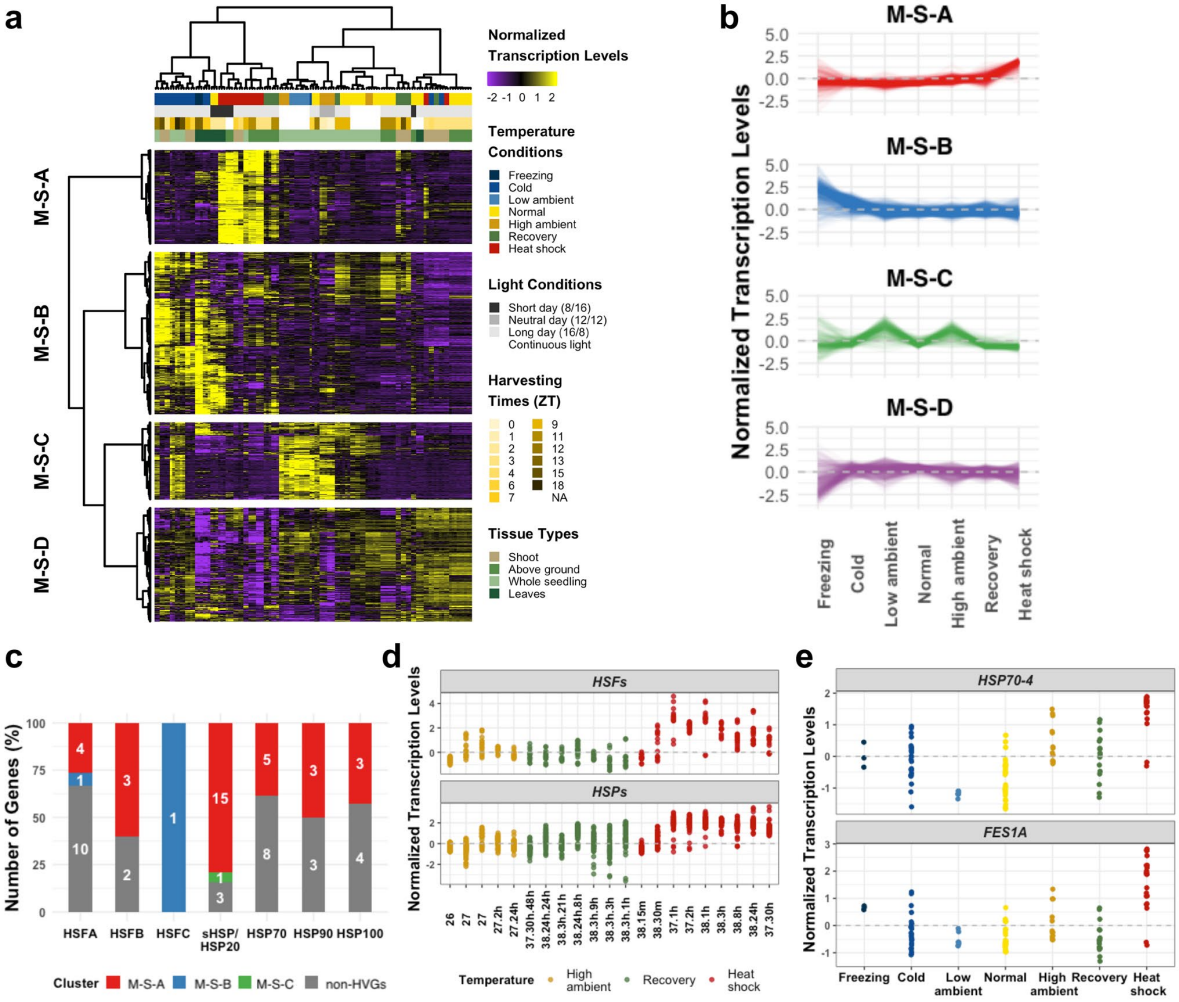
Temperature-responsive genes from the shoot microarray transcriptomes. **a** Overall transcription patterns of the temperature HVGs of the integrated microarray transcriptome obtained from the shoot datasets. **b** Average transcription levels of microarray HVGs in the M-S clusters across different temperature conditions. Each line represents each transcriptomic profile. **c** Distributions of the *HSF* and *HSP* gene families belonging to each M-S cluster. The *HSF* gene families characterized in the *Arabidopsis* genome are *HSFA*, *HSFB* and *HSFC*, and the HSP families are *sHSP/HSP20*, *HSP70*, *HSP90* and *HSP100*. **d**–**e** Distributions of the normalized transcription levels of: **d** seven *HSF* and 26 *HSP* genes in cluster M-S-A from the experiments conducted in the high ambient (orange), recovery (green) and heat shock (red) conditions; **e** *HSP70-4 (AT3G12580)* and *FES1A.* Each dot represents each transcriptomic profile

We first investigated the HVGs of the temperature transcriptomes obtained from the shoot, leaves, above ground tissues and whole seedlings, which would be collectively referred to as “shoot” here for simplicity. Figure 1a demonstrates overall patterns of our integrated microarray temperature transcriptomes from the shoot tissues. The integrated transcriptomes can be hierarchically clusters into four gene sets with unique transcriptional patterns.

#### Cluster M-S-A: genes activated by heat stress

Cluster M-S-A (for Microarray-Shoot-A) consists of 173 HVGs that were highly transcribed in all the heat shock conditions, and to a lesser extent in the heat shock followed by short (1 and 3 h) recovery conditions (Fig. 1a; see Table S4 for a complete list of HVGs). These genes were relatively lowly transcribed in the normal, freezing, low ambient, and even high ambient temperature conditions (Fig. 1b). As expected, the most significant non-redundant GO term in this cluster is “response to heat” (51 genes) (see also Fig. S4a and Table S5 for the complete GO enrichment analysis).

Notable HVGs of Cluster M-S-A are genes in the heat shock factors (HSF) and heat shock protein (HSP) families. At least 21 *HSF* genes (including the *HSFA*, *HSFB* and *HSFC* families) and 45 *HSP* genes (including the *HSP20, 70, 90*, and *100* families) have been annotated in the *Arabidopsis* genome (Swindell et al. 2007). We observed seven *HSFs* (e.g. *HSFA2*, *HSFA7A*) and 26 *HSPs* (e.g. *HSP23.5*, *HSP70-3*, *HSP90-1*) in Cluster M-S-A (Fig. 1c; Table S6). The *HSFs* and *HSPs* in Cluster M-S-A were transcribed at relatively higher levels in the heat shock conditions (> 37 °C), as compared to each of the six other temperature conditions (Fig. S5a, see all the p-values from ANOVA test followed by Tukey’s HSD in Table S2). Looking at the high temperature conditions in more details, the *HSF* and *HSP* HVGs were already mildly transcribed in the heat shock followed by short recovery of 1 or 3 h, and high ambient temperature (25–27 °C) experiments (Fig. 1d, green and orange dots, respectively). When shifted from the normal (24 °C) to heat shock (38 °C) conditions, these genes required up to 1 h after shifting to be fully activated (Fig. 1d, red dots).

One of the well-characterized *HSP* genes directly involved in heat stress responses, *HSP70-4* (Sung et al. 2001), showed significantly higher transcription levels under heat shock (Fig. 1e, see p-values in Table S2). *FES1A* is another interesting HVG in this cluster. The FES1A protein was shown to physically interact with cytosolic HSP70-4 in vivo and in vitro, and prevented degradation of the protein HSP70-4 (Zhang et al. 2010). Both *FES1A* and *HSP70-4* were transcribed at significantly higher levels in the heat shock than other temperature conditions (Fig. 1e), followed by the high ambient temperature, but to a noticeably lesser extent.

#### Cluster M-S-B: genes activated by the cold and freezing conditions

Cluster M-S-B comprises 309 HVGs that were highly transcribed in almost all the freezing (< 0 °C) and cold (3–4 °C) treatments, but not in the low ambient conditions (15–17 °C, Fig. 1a, b). The most enriched GO terms of the cluster are “response to cold” (46 genes), followed by “response to temperature stimulus” (50 genes), and “response to oxygen-containing compound” (79 genes) (Fig. S4b; Table S5).

**Fig. 2:**
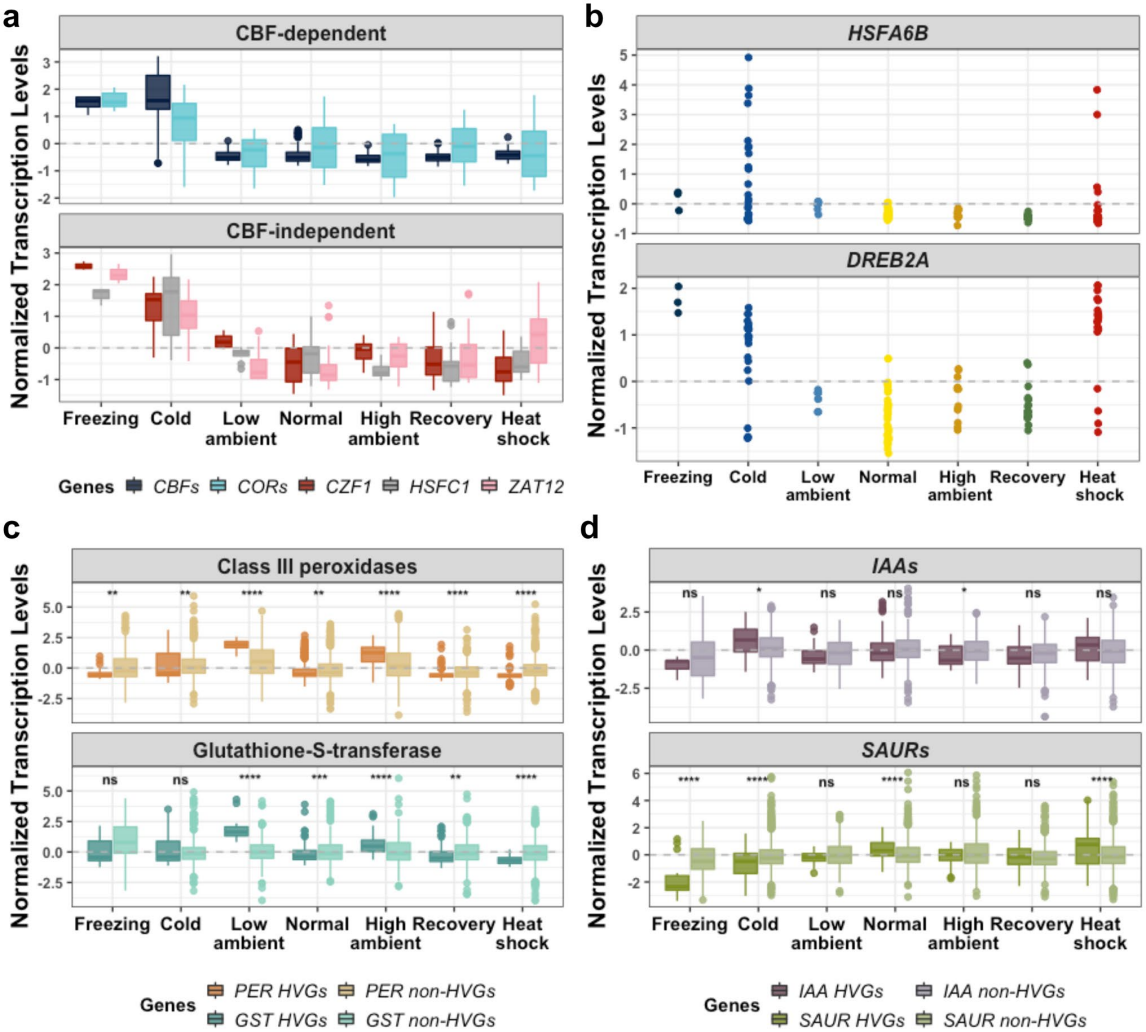
Temperature-responsive genes from the shoot microarray transcriptomes in Clusters M-S-B, M-S-C and M-S-D. **a**–**d** Distributions of the normalized transcription levels of: **a** three *CBF* (*CBF1*, *CBF2 *and* CBF3*) genes, five *COR* (*COR15a*, *COR15b*, *COR27*, *COR47* and *COR413*) genes (upper panel), and *CZF1*, *HSFC1* and *ZAT12* (lower panel) in Cluster M-S-B; **b** *HSFA6B* and *DREB2A*; **c** Ten *PER* and five *GST* HVGs in Cluster M-S-C, in comparison to the rest of 62 *PER* and 48 *GST* non-HVGs in the *Arabidopsis* genome; and **d** Three *IAA* (*IAA1, IAA17* and *IAA29*) and 10 *SAUR* HVGs in Cluster M-S-D, in comparison to the rest of 24 *IAA* and 71 *SAUR* non-HVGs in the *Arabidopsis* genome. Wilcoxon test with Bonferroni correction was performed to statistically assess the differences between the HVGs and non-HVGs; *, **, ***, **** indicate p-values of ≤ 0.05, ≤ 0.01, ≤ 0.001, ≤ 0.0001, respectively. *ns* not significance

The cold temperature response in plants is typically regulated by two major pathways, the *CBF*-dependent and *CBF*-independent pathways (Liu et al. 2019). For the *CBF*-dependent pathway, three *C-REPEAT BINDING FACTOR* (*CBFs*) genes, namely *CBF1-3*, and five *COLD-REGULATED* (*CORs*) genes, namely *COR15a*, *COR15B*, *COR27*, *COR47* and *COR413*, were transcribed highly specifically in the cold and freezing conditions, and they are all the members of Cluster M-S-B (Fig. 2a, see p-values in Table S2). We also observed genes in the *CBF*-independent pathway in this cluster, including *CZF1*, *HSFC1*, and *ZAT12* (Fig. 2a). Interestingly, we also found two *HSF* genes, *HSFA6B* and *HSFC1* in this cluster (Figs. 1c, 2b), suggesting that certain *HSFs* might also be involved in different types of temperature responses, in addition to the well-characterized high temperature conditions. A target gene of the TF HSFC1*, DREB2A* (Huang et al. 2016) is another notable HVG in this cluster (Fig. 2b).

#### Cluster M-S-C: genes activated by ambient temperatures and/or constant light

Cluster M-S-C consists of 156 HVGs that were relatively highly transcribed in ambient or mild temperature treatments, namely the high ambient (25–27 °C) and low ambient (15–17 °C) temperature conditions (Fig. 1a, b). We noted; however, that the experiments under the low ambient temperature treatments in this study were performed under continuous light conditions (Table S1). Thus, we could not rule out the effect of light on the elevated transcriptional levels of Cluster M-S-C HVGs. Along this line, the most enriched non-redundant GO terms are “hydrogen peroxide catabolism” (12 genes), “reactive oxygen species metabolism” (15 genes) and “lipid transport” (13 genes) (Fig. S4c; Table S5).

Indeed, we found that multiple HVGs in Cluster M-S-C belong to the gene families of class III peroxidase (10 genes, see Table S4), glutathione-S-transferase (5 genes) and superoxide dismutase (2 genes). The normalized transcription levels of these HVGs were significantly higher in the low ambient and high ambient temperature conditions, in comparison to the genes in the same families that are not HVGs (Fig. 2c; see p-values from the Wilcoxon test in the figure). *HSP15.4* is the only *HSP* gene belonging to Cluster M-S-C, and its transcriptional level was significantly more elevated under the high ambient temperature as compared to any other conditions (Fig. S5b, p-values in Table S2).

#### Cluster M-S-D: genes suppressed in freezing

Cluster M-S-D contains 217 of all the 855 temperature HVGs from the integrated shoot microarray datasets; however, their transcriptomic patterns across the temperature conditions were less apparent as compared to the three clusters previously described (Fig. 1a), likely due to the lowest variance of transcription levels among its transcriptomic profiles than other clusters (Fig. S4e). Yet, we could still see a clear reduction of relative transcription levels in the freezing conditions (Fig. 1b).

The majority of Cluster M-S-D HVGs fall into general GO terms such as “response to stimulus” (92 genes) and “biological regulation” (69 genes), and notable non-redundant enriched GO terms also include “response to auxin” (20 genes), “pigment biosynthesis” (7 genes) and “regulation of organ growth” (6 genes) (Fig. S4d; Table S5). We observed that the HVGs involved in the “response to auxin” function in this cluster were moderately transcribed in all the temperature conditions investigated, except for freezing where they appeared to be suppressed. This was obvious in two major auxin responsive gene families, consisting of three *INDOLE-3-ACETIC ACID INDUCIBLE* (*IAA*) genes (*IAA1*, *IAA17* and *IAA29*) and 10 *SMALL AUXIN UP RNA* (*SAUR*) genes (*e.g. SAUR14*, *SAUR16*, *SAUR62-63)* (see Table S4). Their normalized transcription levels were markedly reduced under freezing, whereas the non-HVG *IAA* and *SAUR* genes in the genome did not appear to be temperature-sensitive (Fig. 2d).

### Conserved and tissue-specific expression of temperature-responsive genes

Having dissected the integrated transcriptomes of the shoot transcriptome (the “M-S” clusters), here we also looked into the common and unique HVGs identified from the transcriptomes of the roots (“M-R” clusters for microarray of roots). The root microarray transcriptomes were taken from an earlier study (Kilian et al. 2007), consisting of 14 microarray experiments (with duplicates) performed under different temperature profiles: cold (3 °C), heat shock (38 °C), and heat shock and recovery (38 °C, then back to 25 °C).

**Fig. 3:**
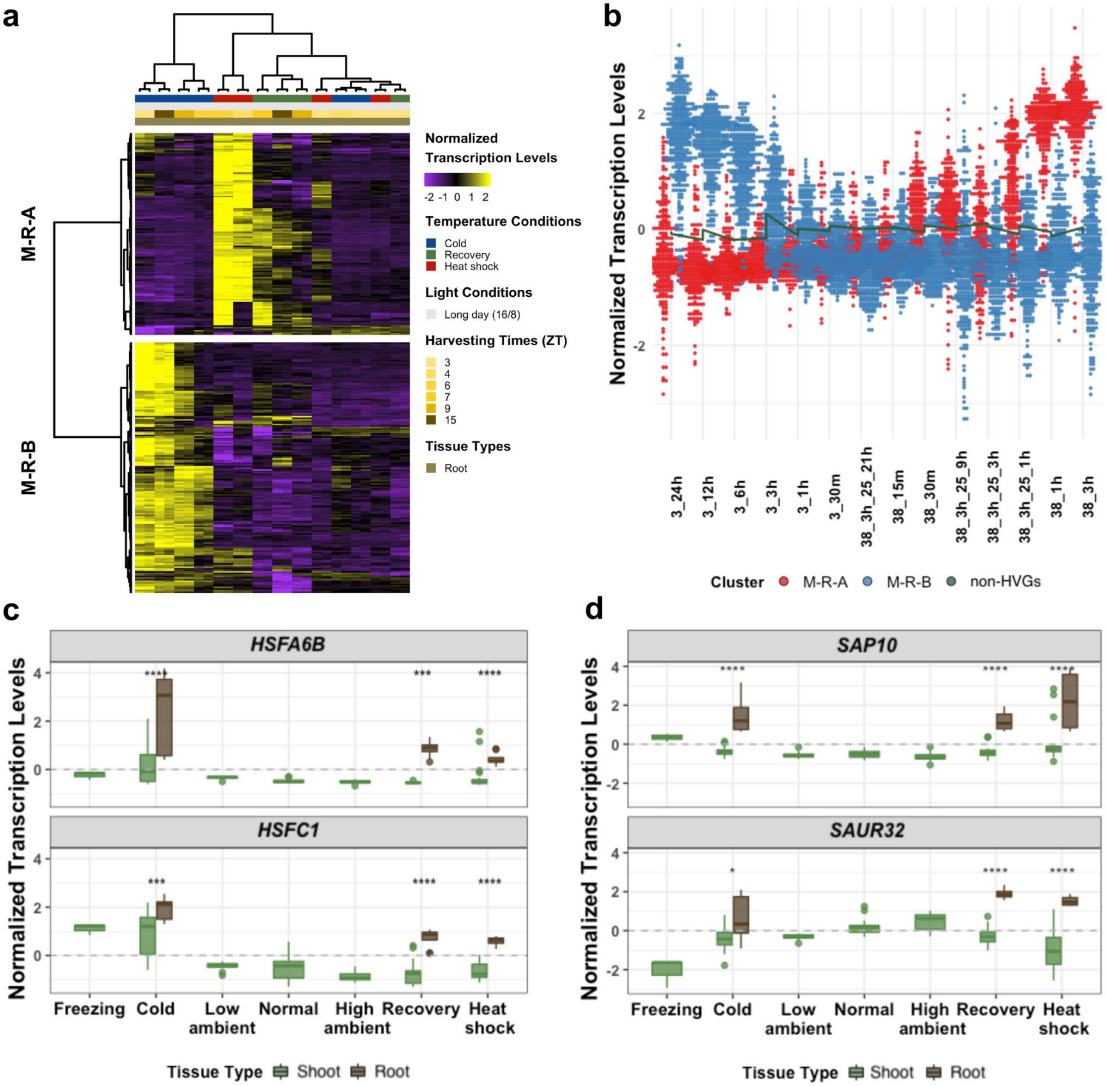
Temperature-responsive genes from the microarray temperature transcriptomes of the shoot and the root. **a** Overall transcription patterns of the temperature HVGs of the root microarray transcriptome obtained from the study by Kilian and colleagues (Kilian et al. 2007). **b** Two distinct transcriptomic profiles of temperature HVGs in the root tissues. Dots represent normalized transcription values of all the HVGs in Clusters M-R-A (red) or M-R-B (blue), while the green line represents the medians of root non-HVGs in corresponding microarray experiments. The temperature profiles were ordered according to the temperatures, the lengths of treatments and recovery periods. **c**–**d** Distributions of the normalized transcription levels of **c**; *HSFA6B* and *HSFC1* and **d** *SAP10* and *SAUR32* in the shoot and root tissues. The transcription levels from the shoot and the root were re-normalized together in order to compare between two tissues. Wilcoxon test with Bonferroni correction was performed to statistically assess the differences between the HVGs of the shoot and the root; *, **, ***, **** indicate p-values of ≤ 0.05, ≤ 0.01, ≤ 0.001, ≤ 0.0001, respectively. *ns* not significance

The 301 HVGs of the root microarray transcriptomic profiles were identified independently to those of the shoot, but using the consistent criteria (see Materials and Methods). The root transcriptomic profiles could be globally grouped into two main clusters: 135 HVGs in Cluster M-R-A (Microarray-Root-A) and 166 HVGs in Cluster M-R-B (Fig. 3a). Cluster M-R-A contains the genes activated by high temperatures (heat shock, and to a lesser extent in the heat shock followed by recovery conditions); while Cluster M-R-B consists of genes largely activated in the cold. Interestingly, the relative transcription levels of HVGs in both Clusters M-R-A and M-R-B appeared to be linked to how long the temperature treatments were applied, as the longer the cold or heat treatments induced higher transcription levels of the HVGs (Fig. 3b). While the HVGs from Cluster M-R-A started to be induced after 30 min of shifting from 24 °C to 38 °C, it took 3 h after shifting from 24 °C to 3 °C for the Cluster M-R-B genes to be induced.

Among the root HVGs in Cluster M-R-A (135 genes), 105 genes (78%) were also the HVGs in Cluster M-S-A, the “high-temperature” cluster of the shoot transcriptomes; whereas 84 (51%) of 166 HVGs in Cluster M-R-B were also the HVGs in Cluster M-S-B, the “low-temperature” cluster (Fig. S6a-b). The most enriched non-redundant GO terms are “response to heat” for Cluster M-R-A (39 genes) and “response to cold” for Cluster M-R-B (28 genes, Table S5), as also seen in Clusters M-S-A and M-S-B, respectively.

We observed five *HSFs* and 23 *HSPs* identified as HVGs in Cluster M-R-A, and the majority are also HVGs in Cluster M-S-A (Table S6). Interestingly, the two *HSF* genes that were activated under low temperature in the shoot (Cluster M-S-B), *HSFA6B* and *HSFC1*, were also identified as low-temperature HVGs in Cluster M-R-B. The two *HSF* genes showed significantly higher transcription levels under the cold temperature condition than other conditions in both tissue types, but higher in the root in comparison to the shoot (Fig. 3c). About a half of HVGs identified in Cluster M-R-B also overlap with those in Cluster M-S-B (Fig. S6b). These include the core cold-responsive genes, *CBF1*, *CBF2* and *CBF3*, and some of the *COR* genes, including *COR15a*, *COR15b* and *COR27* (Fig. S6c).

Looking at the unique HVGs in the root Cluster M-R-A, we observed that the normalized transcription levels of *STRESS* *ASSOCIATED PROTEIN* *10* (*SAP10*) and *SMALL* *AUXIN* *UPREGULATED* *RNA 32* (*SAUR32*) were significantly higher in the root than in the shoot at the same temperature conditions (Fig. 3d, the transcriptomes from the shoot and root experiments were combined and re-normalized).

### Unique regulatory functions of temperature-responsive transcription factors

Having characterized the transcriptionally distinct temperature-responsive genes using existing transcriptomic datasets, in this section we asked how the temperature HVGs can be related to the regulatory functions of key temperature regulators, based on the DNA-binding specificities of publicly available ChIP-seq and DAP-seq profiles. Here, we investigated three TF families known for their regulatory roles in response to temperature changes, namely HSFA1a (Cortijo et al. 2017), PIF4 (Oh et al. 2012), and CBFs (O’Malley et al. 2016). Due to the largest number of transcriptome experiments and the most diverse range of temperature profiles, we focused on the HVGs of the integrated microarray transcriptomes from the shoot (M-S clusters).

#### Transcriptional regulatory roles of HSFA1s under high and low temperature conditions

The HSFA1 TFs are known as one of the key regulatory hubs for heat shock response, but also play an important role in regulating several temperature-responsive genes under various temperature (Cortijo et al. 2017; Liu et al. 2011; Yoshida et al. 2011). Interestingly, the transcription levels of the *HSFA1* family showed only relatively small transcriptional changes across the temperature transcriptomes investigated in this study (Fig. S5e). Here, we re-analyzed the ChIP-seq profiles of HSFA1a obtained from the plant samples grown at 17 °C, 27 °C and 37 °C (Cortijo et al. 2017) and explored their relationship with temperature-responsive genes classified in this study.

**Fig. 4:**
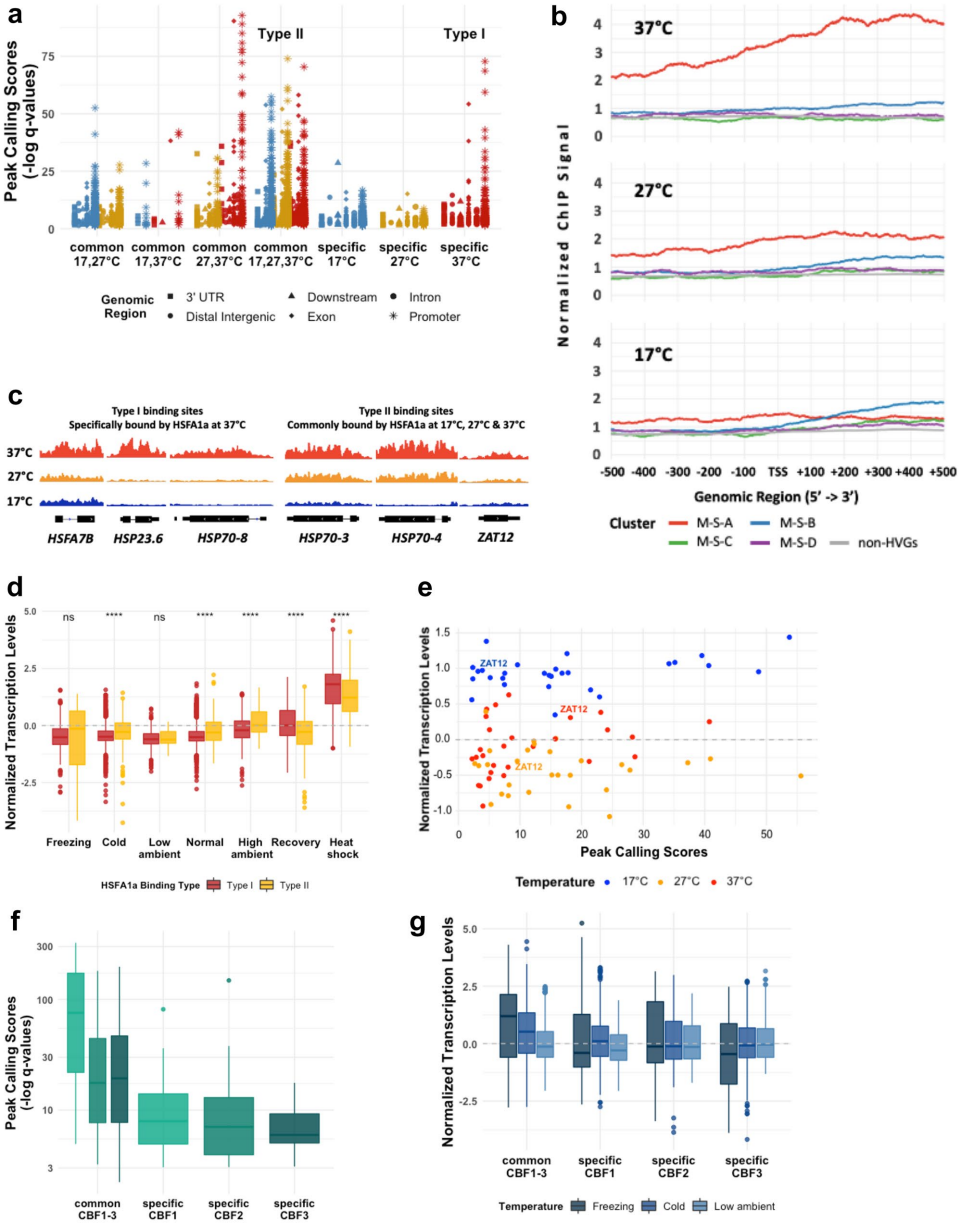
Analyses of DNA-binding occupancies of the HSFA1a, PIF4 and CBFs TFs. **a** Confidence scores from the peak calling of the HSFA1a ChIP-seq experiments obtained at three different temperature conditions. The ChIP results were initially obtained by Cortijo and colleagues (Cortijo et al. 2017) and re-analyzed in this study. **b** Average HSFA1a DNA-binding occupancies at up and downstream to the transcription start sites (TSSs) at 37 °C (top), 27 °C (middle) and 17 °C (bottom) of the temperature-responsive HVGs based on the shoot microarray transcriptomes (M-S clusters). **c** ChIP-seq occupancies of HSFA1a at the selected HSF/HSP target genes: *HSFA7B*, *HSP23.6*, *HSP70-8*, *HSP70-3*, *HSP70-4*, and *ZAT12*. **d** Distributions of the normalized transcription levels of of Type I and II HSFA1a target genes in cluster M-S-A. Wilcoxon test with Bonferroni correction was performed to statistically assess the differences between Type I and II target genes; *, **, ***, **** indicate p-values of ≤ 0.05, ≤ 0.01, ≤ 0.001, ≤ 0.0001, respectively. *ns* not significance. **e** Relationship between peak calling scores and normalized transcription values of the HSFA1a’s target genes in cluster M-S-B across 17 °C, 27 °C and 37 °C. **f** Confidence scores from the peak calling of the CBFs’ DAP-seq (O’Malley et al. 2016) at common and the target genes bound specifically by each of the three CBF TFs. **g** Normalized transcription levels of the target genes common and specific to the three CBF TFs

Overall, the binding sites of HSFA1a were detected with higher confidence scores at the promoter regions of the genes bound only at 37 °C, as compared to the binding sites specific to 17 °C or 27 °C, which might reflect the higher TF occupancies at the promoters of HSFA1a’s target genes in the heat shock condition (Fig. 4a; Table S3, see also Methods). In contrast, the confidence scores of the HSFA1a’s binding sites commonly found in all the three temperatures were in similar ranges (Fig. 4a). This suggests that there might be at least two sets of highly confident HSFA1a binding sites with distinct TF occupancy characteristics: those specifically bound at the heat shock temperature, in this case at 37 °C (Fig. 4a—Type I HSFA1a binding sites herein), and those constitutively bound across these different temperatures (Fig. 4a—Type II HSFA1a binding sites).

We next investigated the relationship of the HSFA1a’s DNA-binding occupancies, and the transcriptomic profiles of the shoot temperature-responsive gene clusters (the M-S clusters). We observed that the binding occupancy of HSFA1a at the proximal DNA sequences to the transcription start site (TSS) of the genes was the highest at 37 °C in Cluster M-S-A (Fig. 4b), whose normalized transcription levels were also elevated the most in heat shock. On the other hand, the average occupancies of HSFA1a bound to the promoters of the M-S-B genes remained largely unchanged between the three temperatures, but still noticeably higher than those at the M-S-C and M-S-D genes, or the non-HVGs. Indeed, the target genes specifically detected at 37 °C (Type I HSFA1a binding sites) were the most enriched binding sites found in M-S-A, (52 of the 107 M-S-A genes, or 49%, p-value < 2.2e-16, Chi-squared test, Fig. S7a; Table S3). For M-S-B, a large proportion of their HSFA1a’s target genes (28 genes out of 92, or 30%) were bound by the TF across all the three temperatures (Type II HSFA1a binding sites; p-value < 2.2e-16*,* Fig. S7b; Table S3).

We next explored the functional enrichment of HSFA1a’s target genes in Cluster M-S-A. As expected, the top three enriched GO terms of the specific HSFA1a’s target genes at 37 °C (Type I) are “response to heat”, followed by “response to temperature stimulus”, and “protein folding”, similarly to the enriched GO terms of Cluster M-S-A itself (Table S7). Among the 52 HSFA1a’s Type I target genes, there are 18 *HSPs* (*e.g. HSP23.6*, *HSP70-8*), and one *HSF* (*HSFA7B*) (Table S6), suggesting that these genes might be regulated by HSFA1a specifically in the heat shock condition (Fig. 4c). Along this line, we also found that the normalized transcription levels of Type I HSFA1a target genes in Cluster M-S-A were significantly higher in heat shock than those of Type II target genes (Fig. 4d, Wilcoxon’s test). In contrast, Type II target genes were transcribed more highly at the high ambient conditions, as compared those of Type I. Exceptions are two *HSPs*, *HSP70-3* and *HSP70-4*, which were identified as the target genes of HSFA1a across the three temperatures, but their confidence scores of the ChIP-seq peak calling were still the highest at 37 °C (Fig. 4c). This might, at least in part, account for the temperature-dependent transcription of the *HSP70-4* gene*,* where its transcription levels were also higher in the heat shock conditions, than in the ambient temperatures (Fig. 1e).

The HSFA1a’s target genes in Cluster M-S-B, which were largely activated at low temperatures, were bound by the TF at 17 °C, 27 °C and 37 °C (Type II HSFA1a binding sites, Fig. 4a; Fig. S7b), and are moderately enriched in the GO term “response to cold” (Table S7). Figure 4e shows that the occupancy levels reflected by the peak calling confidence scores are in the same range across the three temperatures; however, the normalized transcription levels of these HSFA1a’s target genes were relatively higher at the low ambient conditions (approximately 17 °C). *ZAT12*, for instance, described earlier in this study to be up-regulated in the cold as well as heat shock conditions, is an example of an M-S-B gene bound by HSFA1a across 17 °C, 27 °C and 37 °C (Fig. 4c). Other examples of the low temperature-responsive genes constitutively bound by HSFA1a are *ERD14*, *LEA14* and *RD29A*. These together suggest that HSFA1a may also play a partial role in regulating gene transcription at a low ambient temperature, but the binding occupancy of HSFA1a itself does not directly reflect the expression of their target genes and may require additional factors to regulate the transcription.

#### DNA-binding patterns of PIF4 and CBFs and the transcriptional patterns of their target genes

We also investigated the possible links between the temperature transcriptional patterns characterized in this study and the DNA-binding specificities of two other known temperature-integrating TFs, PIF4 (Oh et al. 2012), and CBFs (O'Malley et al. 2016). Unlike HSFA1a, the DNA-binding information of PIF4 and CBFs were not obtained in multiple temperature conditions. Hence, we could only investigate the enrichments of their target genes in our temperature-responsive gene clusters.

As a master regulator of high ambient temperature, several studies have shown the up-regulation of *PIF4* transcription in high ambient temperature (e.g. Koini et al. 2009; Kumar et al. 2012). However, *PIF4* itself was not identified as a HVG in our integrated transcriptome datasets and did not show an elevated normalized transcriptional level in the high ambient conditions (Fig. S8a). This might be due to the limitation of available transcriptomic studies (see Table S1), which were not done at the time of day and light condition that *PIF4* is normally expressed. By re-analyzing the publicly available ChIP-seq profiles of PIF4 of the plants grown at 22 °C (Oh et al. 2012), we investigated its target genes that are temperature HVGs in this study. We identified 162 PIF4’s target genes that overlap with our M-S HVGs. The transcription patterns of the PIF4’s target genes in all the four temperature-responsive M-S clusters appeared to be similar to the overall patterns of all the genes in each cluster (Fig. S7c). Cluster M-S-D is the most enriched PIF4-target cluster (64 out of 162 PIF4’s target genes), possibly reflecting the fact that PIF4 mediates thermomorphogenesis growth via auxin, and that “response to auxin” is one of the most enriched GO terms of M-S-D (see p-values in Table S7).

CBFs are known regulators of cold stress (Liu et al. 1998), and their transcriptions can be induced by both the low ambient and cold temperature conditions (Dong et al. 2020; Novillo et al. 2004). By re-investigating the DAP-seq profiles of CBF1-3 (O'Malley et al. 2016), we could predict 227, 130 and 214 temperature-HVGs that are the putative target genes of CBF1, CBF2 and CBF3, respectively (Fig. S7d). As expected, the most enriched target genes for all CBF TFs are in Cluster M-S-B and the most enriched GO term is “response to cold” (Table S7). In our integrated transcriptomic datasets, *CBFs* themselves were also characterized as HVGs in Cluster M-S-B, and their transcription levels were up-regulated under low temperature conditions, similarly to their target genes in Cluster M-S-B (Fig. S7e). In addition, the common binding sites predicted for all the three CBFs had higher peak calling confidence scores (Fig. 4f), which may infer “strong” CBF binding sites. Interestingly, the direct target genes downstream to the common CBF binding sites were more highly induced under the freezing and cold conditions, in comparison to those bound by only one CBF (Fig. 4g; see p-values from Wilcoxon test in Fig. S7f). This finding provides evidence supporting previous studies that CBFs play an essential role under low temperature conditions and have partial functional redundancy when regulating cold-responsive genes (Jia et al. 2016; Zhao et al. 2016).

## Discussion

### Global analysis of integrated transcriptomic profiles provides an overview of temperature-responsive transcription patterns

Temperature is one of the major environmental factors controlling plant’s growth and developmental processes. To date, thanks to the advances in omic technologies, there have already been a large number of studies that employed high-throughput gene expression profiling to investigate the overall effect of different temperature conditions on the global gene expression patterns in plants (e.g. Cortijo et al. 2017; Dickinson et al. 2018; Higashi et al. 2015; Kilian et al. 2007). However, to the best of our knowledge, there is yet a comparative study that integrates multiple temperature transcriptomes and dissects the conserved and specific genes and pathways that are involved in the plant’s responses to different types of temperature conditions.

In this study, we have taken advantage of the publicly available transcriptomic data of *A. thaliana* grown under a wide range of temperatures, both from microarray (153 transcriptomic profiles, 66 unique experiments, and seven types of temperature conditions), and RNA-seq (106 transcriptomic profiles, 73 unique experiments, three types of temperature conditions), integrated and analyzed them together in a single study (Table S1; Fig. S1).

We took a top-down approach to globally identify all the genes whose transcription levels are highly influenced by different temperature conditions or treatments, which we termed highly variable genes, or HVGs. Unlike the conventional analysis of differentially expressed genes (DEGs), where the significance of differential expression is statistically assessed between two specific conditions or treatments, HVGs in this study represent the genes whose transcription patterns are highly variable across multiple temperature conditions, not only between the given two conditions. In total, here we have characterized four HVG clusters with distinct transcription patterns of the shoot microarray dataset (the M-S clusters), two clusters from the root microarray dataset (M-R clusters); and in Supplementary Results, three clusters from the seedling RNA-seq dataset (R-S clusters), and two clusters from the root RNA-seq dataset (R-R clusters).

We carefully integrated multiple transcriptomes and re-normalized them to mitigate the biases between the studies and batches, and unbiasedly characterized HVG clusters based on the correlations of their transcriptional patterns across the available temperature conditions, regardless of their previously characterized functions. Similar approach has also been taken by Shen and coworkers (Shen et al. 2017), who combined microarray abiotic stress datasets and successfully identified common and specific gene modules to different abiotic stresses. Such comparative transcriptomic analyses have several advantages over conventional meta-analysis studies, which normally analyzed the DEGs from different studies separately and compared the gene lists at the end, especially in terms of higher sensitivity of detecting differentially expressed gene sets enrichment (Kosch and Jung 2019).

### Diverse transcriptional profiles of HSFs and HSPs in multiple temperature conditions

HSPs are known to be responsible for mitigating protein misfolding under various stresses including heat (Jacob et al. 2017; Park and Seo 2015), and they themselves are regulated by the HSF TFs (Jacob et al. 2017; Nover et al. 2001). At least 21 *HSF* genes (including the *HSFA*, *HSFB* and *HSFC* families) and 45 *HSP* genes (including the *HSP20*, *70*, *90*, and *100* families) have been annotated in the *Arabidopsis* genome (Swindell et al. 2007). By carefully investigating the clusters of HVGs with transcriptionally unique patterns across different temperature conditions, we observed diverse transcriptional profiles, and potentially functional roles of the *HSF* and *HSP* genes (Tables 1 and S6; Fig. 1c).

**Table 1:** Characteristics of temperature-responsive genes, their transcription patterns, tissue-specific transcriptions, and temperature TF binding patterns

Gene family/group	Transcription patterns	Tissues/clusters	Binding patterns of temperature TFs: HSFA1a, PIF4, CBFs)	Key genes	Remarks
Heat shock factor (HSF)	Up highly at heat shock and moderately at ambient high temperatures	Shoot and root (M-S-A, M-R-A)	Bound by HSFA1a at high ambient (27 °C) and heat shock (37 °C) temperatures, and with the highest peak-calling confidence scores at 37 °C	*HSFA2*, *HSFA7A*, *HSFB2A*, *HSFB2B*	Conserved high temperature-responsive genes in the shoot and the root
			Bound by HSFA1a at heat shock temperature (Type I binding sites)	*HSFA7B*	
	Up highly at freezing and cold temperatures; higher in the root than the shoot	Shoot and root (M-S-B, M-R-B), higher in the root	Not targets of these TFs	*HSFC1*	CBF-independent cold responsive pathway
	Up highly at cold and prolonged heat shock temperatures; higher in the root than the shoot			*HSFA6B*	Might be involved in cold temperature-responsive functions through *DREB2A*
Heat shock protein (HSP)	Up highly at heat shock and moderately at ambient high temperatures	Shoot and root (M-S-A, R-S-B, M-R-A, R-R-A)	Bound by HSFA1a at low ambient, high ambient and heat shock temperatures (Type II), and with the highest confidence scores in heat shock	*HSP18.5*, *HSP23.5*, *HSP90-1*	Conserved high temperature-responsive genes in the shoot and the root
		Shoot and root (M-S-A, R-S-B, R-R-A)		*HSP70-4*	
		Shoot and root (M-S-A, R-S-B, M-R-A)		*HSP70-3*	
			Bound by HSFA1a at heat shock temperature (Type I)	*HSP70-8, CLPB3*	
		Shoot and root (M-S-A, M-R-A)	Bound by HSFA1a at heat shock temperature (Type I)	*HSP70-5*	
	Up at ambient temperature under light	Shoot (M-S-C)	Not targets of these TFs	*HSP15.4*	
	Up highly at heat shock	Root (M-R-A)	Bound by HSFA1a at low ambient, high ambient and heat shock temperatures, and with the highest confidence scores in heat shock	*HSP70-7*	
C-repeat binding FACTORS (CBFs)	Up at low temperatures	Shoot and root (M-S-B, M-R-B)	CBF1 is bound by CBF1 itself CBF2 is bound by HSFA1a at 17 °C	*CBF1*, *CBF2*, *CBF3*	
COld -responsive (COR)	Up at low temperatures	Shoot and root (M-S-B, M-R-B), higher in shoot	Bound by at least one CBF	*COR15a*, *COR15b*	CBF-dependent cold responsive pathway
		Shoot (M-S-B)	Bound by all the CBFs, and also constitutively bound by HSFA1a at low ambient and heat shock temperatures	*COR47*	
Zinc-finger protein	Up highly at low temperatures and moderately at heat shock	Shoot (M-S-B)	Bound by PIF4, CBF1-3, and HSFA1a at low ambient, high ambient and heat shock temperature (Type II)	*ZAT12*	Identified as a “general” stress-induced genes (Kilian et al. 2007) CBF-independent pathway
	Up highly at cold and heat shock temperatures	Root (M-R-A)	Not targets of these TFs	*SAP10*	
Auxin-related genes	Low at freezing	Shoot (M-S-D)	Most are bound by PIF4	*IAA1*, *IAA17*, *IAA29*, *SAUR14*, *SAUR16*	
	Up at heat shock	Root (M-R-A)	Not targets of these TFs	*SAUR32*	
Other common temperature genes between the shoots and roots	Up at high temperature	Shoot and root (M-S-A, R-S-B, M-R-A, R-R-A)	Bound by HSFA1a at high ambient and heat shock temperatures, and with the highest confidence scores in heat shock	*DNAJ*, *FKBP65*, *HOP3*, *SGT1a*, *MBF1C*, *SR30*, *SR45A* and *AT5G12110*	Conserved high temperature-responsive genes in the shoots and roots

Overall, the vast majority of *HSF* and *HSP* HVGs are in Cluster M-S-A (Fig. 1c), except for two *HSFs* (*HSFC1* and *HSFA6B*) in Cluster M-S-B; and one *HSP* (*HSP15.4*) in Cluster M-S-C. The *HSFs* and *HSPs* in Cluster M-S-A were more activated in heat shock, as compared to the high ambient and other temperature conditions (Fig. S5a). The increased transcription levels of *HSFs* and *HSPs* in Cluster M-S-A were partially observed in the heat shock followed by short recovery and high ambient temperature (25–27 °C) experiments, and these genes were fully activated at 1 h after the heat shock treatment (Fig. 1d). This suggests that there might be thresholds of the temperature and the length of treatment for these *HSFs* and *HSPs* to be activated.

When we compared the *HSF* and *HSP* HVGs identified in the high temperature clusters from different tissues and transcriptomic methods (Clusters M-S-A, R-S-B, M-R-A and R-R-A), there are three *HSP* genes commonly identified as HVGs in all the four analyses, namely *HSP18.5*, *HSP23.5* and *HSP90-1* (Table 1). As these core members of the *HSP* family were consistently activated in every tissue, they serve as excellent candidates for the universal biomarkers of high temperature responses in plants.

Despite being generally known for their regulatory roles under high temperatures, we also observed two *HSFs* with prominent transcription induction at low temperature. *HSFA6B* and *HSFC1* were classified as “low-temperature” HVGs in the shoot (Cluster M-S-B, Fig. 2a, b) and root transcriptomes (Cluster M-R-B, Fig. 3c), but their transcription levels appeared to be more prominent in the root (Fig. 3c). A previous study showed that transcription of *HSFC1* was inversely correlated to high temperature treatments, as its transcription decreased after 1 h of shifting *Arabidopsis* from 21 °C to 37 °C (Guan et al. 2014). It has also been shown to be one of the first-wave TFs that were induced under cold temperature exposure (Park and Seo 2015; Zhao et al. 2016). The *HSFC1* transcription in the *cbf123* triple mutant plant shifted to cold (4 °C) for 1 h was still transiently up-regulated, whereas the CBF-dependent cold-responsive genes showed very little change in expression, suggesting that *HSFC1* could be activated at a low temperature by a CBF-independent pathway (Zhao et al. 2016). For *HSFA6B*, we found no previous evidence that the gene is differentially transcribed under cold temperature, although it has been shown to directly bind to the heat shock elements (HSEs) in the promoter of *DREB2A* (Huang et al. 2016)*,* another HVG in Cluster M-S-B, and activate its transcription (Fig. 2b), suggesting that *HSFA6B* might be involved in cold temperature-responsive functions through *DREB2A*.

For the rest of the *HSF* and *HSP* genes (e.g. *HSFA1s*, *HSP70-6*, *HSP90-5*) that did not pass the cut-off for HVGs, we observed that their transcription levels also slightly varied across the temperature conditions, but at relatively smaller extents as compared to other HVGs in Cluster M-S-A (Fig. S5e). Despite the HSFA1 TFs themselves not being HVGs, their target genes such as *HSFA2*, *HSFA3* and *HSFA7s* were HVGs in Cluster M-S-A. It might be possible that the upstream TFs regulating heat stress signaling cascades require only small transcriptional changes to be sufficient for amplification of the cellular signaling through the downstream heat-responsive genes, as suggested earlier (Cortijo et al. 2017).

### Tissue-specific high temperature-responsive genes

We observed two HVGs that were predominantly transcribed in the root at high temperatures (Cluster M-R-A), *SAP10*and *SAUR32* (Fig. 3d; Table 1), suggesting their might have specific function in roots under high temperatures. A previous study has shown that SAP10 was expressed predominantly in roots and floral parts, and that overexpression of *SAP10* could rescue *Arabidopsis* from heat stress (Dixit and Dhankher 2011). For *SAUR32,* to the best of our knowledge, there has been no earlier evidence of its involvement in plant thermomorphogenesis. For the HVGs found all the high temperature clusters (Clusters M-S-A, R-S-B, M-R-A and R-R-A), in addition to the three *HSP* genes described in the previous section (*HSP18.5*, *HSP23.5* and *HSP90-1*), we also found eight other HVGs shared by all the high temperature clusters, namely *DNAJ, FKBP65*, *HOP3*, *SGT1a*, *MBF1C*, *SR30*, *SR45A* and *AT5G12110* (Table 1).

### Cold-responsive genes in dependent and independent to the CBF families

We observed that the normalized transcription levels of the three *CBF* HVGs in the shoot and root samples (Clusters M-S-B and M-R-B) were high in the cold and freezing conditions (Figs. 2a and S6c; Tables 1 and S2), as previously observed in plants subjected to sub-zero acclimation (Le et al. 2015). Similar pattern was observed in the five *COR* HVGs, but the *COR* genes appeared to be expressed higher in the shoot than the root (Fig. S6c). *CBFs* are one of the most well-characterized genes regulating cold acclimation and freezing tolerance (Jaglo-Ottosen et al. 1998; Medina et al. 2011). There are four *CBF* genes characterized in the *A. thaliana* genome, namely *CBF1*, *CBF2*, *CBF3* and *CBF4*, or also known as *DREB1B*, *DREB1C* and *DREB1A* and *DREB1D*, respectively (Mizoi et al. 2012). The cold-inducible *CBFs,* namely *CBF1*, *CBF2* and *CBF3,* are all presented in Clusters M-S-B and M-R-B; whereas *CBF4*, which was not characterized as a temperature HVG in this study, has been shown to be induced by the drought and ABA-treatment (Haake et al. 2002).

CBF1-3 act as the TFs that regulate other cold-responsive genes, including the *COR* genes (Fowler and Thomashow 2002). There are five *COR* genes identified as HVGs in Cluster M-S-B, namely *COR15a*, *COR15b*, *COR27*, *COR47* and *COR413*. During freezing, the COR15a and COR15b proteins have been demonstrated to help stabilize the inner membrane of chloroplast (Navarro-Retamal et al. 2018). For *COR47*, its overexpression could enhance freezing tolerance (Puhakainen et al. 2004), and the COR47 protein accumulation was induced under 4 °C (Nylander et al. 2001) and might contribute to cryoprotective activity by its hydrophobic amino acid residuals (Ohkubo et al. 2020). However, to the best of our knowledge, it is not yet known how *COR27* and *COR413* are related to cold responses.

Cold responsive genes can also be regulated by the CBF-independent pathway, whose members include *HSFC1*, *ZAT12* or *CZF1* (Jia et al. 2016; Park et al. 2015). These three genes were identified as HVGs in Cluster M-S-B and were transcribed at significantly higher levels under cold and freezing conditions than almost all other conditions (Fig. 2a; p-values in Table S2). An interesting exception is *ZAT12*, as its normalized transcription levels were elevated not only under cold and freezing, but also in heat shock (Fig. 2a, pink boxplots; p-values in Table S2). This is in line with an earlier study showing up-regulation of *ZAT12* at 4 °C and 38 °C, as well as other abiotic stresses such as oxidative and salinity stresses (Davletova et al. 2005).

### Distinct DNA-binding patterns of temperature-responsive TFs may define transcriptional outcomes

**Fig. 5:**
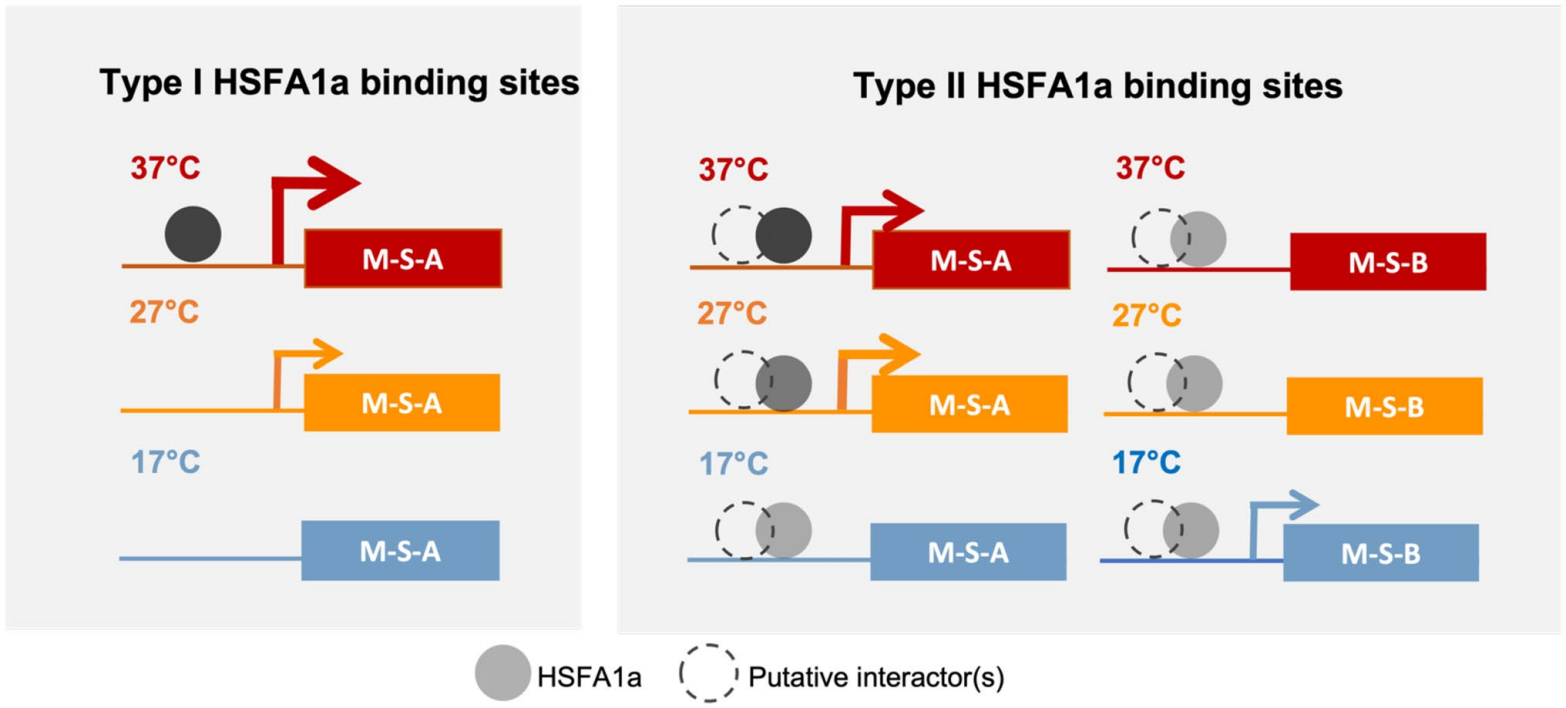
A working model of how HSFA1a regulates the high and low temperature-responsive genes. The darker grey colors represent the higher the confidence scores of the HSFA1a peak calling, which might reflect the DNA-binding occupancy of the TF

HSFA1a is known as a master regulator of heat shock (Liu and Charng 2012; Ohama et al. 2017) as well as of the high ambient temperature conditions (Cortijo et al. 2017). Yet, we found that the transcription level of the TF itself was not highly induced in heat shock and thus was not identified as a HVG (Fig. S5a). However, its target genes were not only highly enriched in the high temperature clusters (M-S-A and M-R-A), but also to our surprise, in the low temperature clusters in certain cases (M-S-B, Fig. S7a, b). Remarkably, the genes induced by high temperature, including a large proportion of Cluster M-S-A HVGs such as *HSFA7B, HSP23.6* and *HSP70-8*, were identified as the target genes of HSFA1a specifically in heat shock (37 °C, Fig. 4c—Type I binding sites and Fig. S7a), and their transcription levels were also highest in the heat shock condition (Figs. 4d, 5). Interestingly, some M-S-A genes were predicted as the target genes of HSFA1a at the low ambient (17 °C), high ambient (27 °C) and heat shock (37 °C) temperatures (Fig. 4c—type II binding sites), and they tended to be transcribed more highly at the high ambient temperature than those of type I HSFA1a binding sites (Fig. 4d). As the binding occupancy at type II target genes does not directly reflect their transcriptional levels, additional interacting factors are likely required to mediate this temperature transcriptional specificity.

Looking into the target genes of HSFA1a that were induced at low temperature, which were mainly HVGs in Cluster M-S-B such as *ZAT12,* they appeared to be constitutively bound by HSFA1a at the low, high ambient, and heat shock temperatures (Fig. 4b—blue line; Fig. 4c—Type II binding sites; Table 1). The heat stress regulator HSFA1 has already been demonstrated for its role in mediating cold responsive pathways by Olate and colleagues (Olate et al. 2018). The authors shown that NON-EXPRESSER OF PATHOGENESIS-RELATED GENE 1 (NPR1), a master regulator in a pathogenic-responsive pathway, could interact with HSFA1s and activate the transcription of cold-induced heat shock-responsive genes, to promote cold acclimation in *Arabidopsis* (Olate et al. 2018). It is not clear; however, if the regulatory function of the HSFA1 family in mediating the crosstalk between heat and cold responsive mechanisms is strictly through NPR1, or also seen in other cold responsive pathways.

Here, we proposed a working model of the multiple roles of *HSFA1a* on regulating the temperature-responsive HVGs in Fig. 5, whereby the HSFA1a TF can take up different DNA-binding configurations at different temperatures: type I for specific binding in the heat shock condition, and type II for constitutional binding across the low ambient, high ambient, and heat shock conditions. We hypothesized that these distinct DNA-binding patterns of HSFA1a might be one of the mechanisms that determine the transcription levels of its target genes. The type I HSFA1a binding sites were predominantly found at the promoters of the high temperature Cluster M-S-A HVGs, while the type II binding sites were seen in both Cluster M-S-A and the low temperature Cluster M-S-B. Only at the heat shock target genes that the levels of HSFA1a binding occupancies reflect the downstream transcription levels, whereas the constitutional HSFA1a binding seen at the high and low temperature target genes do not (Figs. 4d, e and 5). This suggests that other interacting factors might be required to determine the transcription levels of the HSFA1a target genes in non-heat shock conditions.

For the low temperature TFs, CBF1-3 more likely occupy the binding sites that are shared by all the three CBFs, suggesting their functional cooperativity and redundancy under low temperature conditions (Fig. 4f). Intriguingly, these strong CBF bindings are linked to higher levels of transcriptional induction under the freezing and cold temperatures (Fig. 4g). In addition to the Type I and II binding sites of HSFA1a described above, this provides another line of evidence that the DNA-binding configurations can potentially be used as predictors of transcriptional responses of the target genes.

## Conclusion and future perspectives

We have demonstrated how a large-scale comparative transcriptomic analysis can provide a bird-eye view of the global transcriptional patterns of the model plant *Arabidopsis* grown under diverse temperature ranges, covering the freezing, cold, low and high ambient, and heat shock conditions. We combined the transcriptomic profiles from multiple studies and carefully normalized them altogether, to mitigate technical biases when possible. Using our high-quality integrated transcriptomic dataset, we were able to investigate the influences of multiple temperature conditions and treatments simultaneously, as well as explore the conserved and condition-specific temperature-responsive genes to different environmental temperature conditions.

We note; however, that such analysis is inevitably confounded by the availability of publicly available transcriptomic datasets, which might not cover all the factors that may also influence the transcription levels, in addition to the temperature responses. With this in mind, we carefully dissected and documented the details of growth conditions of the integrated transcriptomic profiles. We also performed thorough statistical tests to ensure that the influences between certain conditions of interest are at least statistically significant, despite the potential effects from other environmental conditions. In certain cases, we could not completely rule out the interplay between temperatures and other environmental factors, such as light, photoperiod, and diurnal expression.

All in all, we have carefully characterized and documented a number of clear and directly testable hypotheses of the temperature-responsive genes that demonstrated unique and conserved transcriptional patterns among the temperature conditions and plant tissues (Table 1). These genes serve as prospective candidates for in-depth experimental validations. The integrated dataset presented in this study also serves as a useful resource for in-depth temperature-specific gene expression analyses of known and novel temperature-responsive genes in *Arabidopsis*, and potentially their homologs in other model plant species.

## Supplementary Information

Below is the link to the electronic supplementary material.

Supplementary file1 (PDF 1225 kb)

Supplementary file2 (PDF 3948 kb)

Supplementary file3 (XLSX 27 kb)

Supplementary file4 (XLSX 24 kb)

Supplementary file5 (XLSX 464 kb)

Supplementary file6 (XLSX 2238 kb)

Supplementary file7 (XLSX 9795 kb)

Supplementary file8 (XLSX 54 kb)

Supplementary file9 (XLSX 5214 kb)

## Data Availability

Data are included as electronic supplementary materials.
