# Supplementary material for: Large-scale comparative transcriptomic analysis of temperature-responsive genes in Arabidopsis thaliana
Source: Plant Mol Biol. 2022 Jan 1;110(4-5):425–43. doi: 10.1007/s11103-021-01223-y (PMC9646545; doi:10.1007/s11103-021-01223-y)
Supplement: Supplementary file 1 — Supplementary file1 (PDF 1225 kb) [file 11103_2021_1223_MOESM1_ESM.pdf]

SUPPLEMENTARY RESULTS

Large-scale comparative transcriptomic analysis of temperature-responsive genes in *Arabidopsis thaliana*

Napaporn Sriden[1,2] and Varodom Charoensawan[2,3,4,*]

[1] Doctor of Philosophy Program in Biochemistry (International Program), Faculty of Science, Mahidol University, Bangkok 10400, Thailand.

[2] Department of Biochemistry, Faculty of Science, Mahidol University, Bangkok 10400, Thailand

[3] Integrative Computational BioScience (ICBS) Center, Mahidol University, Nakhon Pathom 73170, Thailand

[4] Systems Biology of Diseases Research Unit (SyBID), Faculty of Science, Mahidol University, Bangkok 10400, Thailand

*Corresponding author. E-mail: varodom.cha@mahidol.ac.th

Supplementary Methods

Transcriptomic data pre-processing and analyses for RNA-seq analyses

The fastq files of the *Arabidopsis thaliana* ecotype *Col-0* transcriptomes subjected to different temperature conditions were downloaded from the Sequence Read Archive (SRA), and the integrated dataset comprises 106 individual files from 73 unique experiments (see Table S1). After being quality controlled using FastQC (http://www.bioinformatics.babraham.ac.uk/projects/fastqc), the raw reads were trimmed using Trimmomatics (Bolger et al. 2014) if the per-base sequence quality was lower than 28. Cleaned reads were mapped to the TAIR10 *Arabidopsis thaliana* genome using HISAT2 (Kim et al. 2019), and the optical duplicates were removed using Picard (https://github.com/broadinstitute/picard), as previously described (Cortijo et al. 2017; Cortijo et al. 2018). The aligned reads of all the combined RNA-seq datasets were normalized using the EstimateSizeFactor function in the DESeq2 package (Love et al. 2014), to also mitigate the biases between different batches and experiments. Lowly transcribed genes, including those with the normalized read counts lower than one across at least five RNA-seq transcriptomes were discarded (Fig. S2a), resulting in 25,099 genes used in subsequent analyses.

Identification of highly variable genes (HVGs) across the temperature profiles for RNA-seq analyses

We followed the same pipeline used for the microarray dataset to identify RNA-seq HVGs, except for the cut-offs: -log p-value > 10 and SD > 250 for the seedling RNA-seq dataset; -log p-value > 3 and SD > 250 for the root RNA-seq dataset (see Fig. S2 for inflexion points).

Gene ontology (GO) enrichment analysis for RNA-seq HVGs

GO term enrichment analysis was carried out as for the microarray dataset, except that the genes annotated in the TAIR10 *Arabidopsis thaliana* genome were used as the reference background.

Supplementary Results

RNA-seq transcriptomic profiles reveal the dynamics of temperature-responsive genes throughout the day

In addition to the temperature transcriptomes from microarray experiments, we also explored the temperature HVGs using publicly available RNA-seq transcriptomes. Overall, the RNA-seq temperature transcriptomic experiments were performed in fewer and less diverse temperature conditions than those of the microarray, namely low ambient (15°C – 17°C), normal (20°C - 23°C) and high ambient (27°C) temperatures. Taking into account the tissue-specific transcription patterns (Fig. S3b), we performed the analysis of the RNA-seq profiles of the whole "seedlings" (R-S clusters) and the "root" (R-R clusters) separately (Table S1). The plant samples of our integrated RNA-seq profiles were harvested at different time points throughout the day, making them particularly suitable for investigating the diurnal dynamics of temperature transcriptional responses. The three hierarchically clustered HVGs of the seedling RNA-seq were as follows (Fig. S9a).

Cluster R-S-A: genes activated by high ambient temperature during nighttime

Cluster R-S-A (RNA-seq-Seedlings-A) consists of 499 temperature HVGs whose expressions were not only temperature-dependent, but also time-of-day-dependent (Fig. S9a-b; see Table S4 for the list of HVGs). We observed significantly elevated normalized transcription levels of the HVGs specifically in the high ambient (27°C, Fig. S9b, orange boxes) and normal (20°C – 23°C, yellow boxes), as compared to the low ambient (15°C – 17°C, blue boxes) conditions; and to a larger extent at nighttime and dawn (ZT16, ZT20, ZT22 and ZT0, Fig. S9b; see Table S2 for p-values from Tukey's HSD, ZT0 – the Zeitgeber time, when the light was on). The most enriched non-redundant GO terms of this cluster is "response to light" (46 genes), where their transcription

levels under high ambient temperature were also higher than other temperatures during nighttime (Fig. S9c; Table S2). Examples of the HVGs of Cluster R-S-A were *CRY1*, *PHYA, PIF5* and *LHY* (see Table S4).

Cluster R-S-B: genes activated by high ambient temperature during daytime

Cluster R-S-B comprises 271 temperature HVGs that appeared to be slightly activated during the daytime, and more elevated by the high ambient temperatures at ZT1 (Fig. S9a-b). Indeed, we found 25 HVGs associated with the "response to heat" function in this cluster, including 14 *HSP* genes (e.g. *HSP18.5*, *HSP70-3* and *HSP90-1*, see Table S6).

Focusing on the transcriptional patterns of the *HSP* genes, the activation by the high ambient temperature was even more apparent at ZT1 and ZT2, while the differences between the temperatures were indistinguishable at other time points, especially at night (Fig. S9d; p-values in Table S2). This fall in line with the morning peak expression of the *HSP* genes under high ambient temperature previously observed (Dickinson et al. 2018).

Cluster R-S-C: genes activated by low ambient temperature

Cluster R-S-C consists of 231 temperature HVGs showing transcriptional activation under the low ambient temperatures (15°C-17°C) throughout the day (Fig. S9b, blue boxes). Unlike the low temperature cluster of the microarray dataset (Cluster M-S-B), the HVGs of Cluster R-S-C are not specifically enriched in the genes with known cold-related functions, likely because these RNA-seq profiles were performed at only low ambient temperature, instead of the cold and freezing temperatures as in the microarray profiles. In fact, we observed only two *COR* genes, *COR15B* and *COR27,* in this cluster. Strikingly, the normalized transcription levels of these two *COR* genes were high at ZT8-12 (Fig. S5c-d). In addition, we observed 23 ribosome-encoding genes in Cluster R-S-C, leading to the enrichment of the GO term "translation" (Table S5). Hence, it appeared the translation-related HVGs in this cluster were activated by the low ambient temperatures (15°C-17°C) (Fig. S9e, p-values in Table S2). This falls in line with an earlier study demonstrating the increases in protein levels and ribosome contents in fresh weight of *Arabidopsis thaliana* grown under constant low ambient temperatures (12°C-16°C), as compared to those grown at 24°C (Pyl et al. 2012).

Temperature-responsive genes in the root from the RNA-seq experiments

For the root RNA-seq transcriptome (Martins et al. 2017), we identified 358 root RNA-seq HVGs, which could be grouped into two main clusters based on the transcription patterns. Cluster R-R-A (RNA-seq-Root-A) consists of 157 genes that were activated under high ambient temperature (26°C), while Cluster R-R-B consists of 201 genes that were repressed under the high ambient temperature, as compared to the moderate temperature (21°C, Fig. S6d). The most enriched non-redundant GO term in Cluster R-R-A is "response to heat" (12 genes, Table S5). High ambient temperature could activate transcription of the *HSF* and *HSP* genes, but to the lesser extent to heat shock, and we found six *HSP* genes, namely *HSP18.5*, *HSP23.5*, *HSP70-2, HSP70-4, HSP90-1* and *CLPB1* in Cluster R-R-A (Fig. S6e; Table S6). For the moderate temperature cluster, Cluster R-R-B, we did not observe the *CBF* or *COR* genes, most likely because 21°C was not low enough to activate the cold-responsive genes, as the *CBF* transcripts typically become detectable at 14°C or lower (Zarka et al. 2003).

Supplementary References

Bolger AM, Lohse M, Usadel B (2014) Trimmomatic: a flexible trimmer for Illumina sequence data. Bioinformatics 30:2114-2120. https://doi.org/10.1093/bioinformatics/btu170

Cortijo S, Charoensawan V, Brestovitsky A, Buning R, Ravarani C, Rhodes D, Noort JV, Jaeger KE, Wigge PA (2017) Transcriptional regulation of the ambient temperature response by H2A.Z nucleosomes and HSF1 transcription factors in *Arabidopsis*. Mol Plant 10:1258-1273. https://doi.org/10.1016/j.molp.2017.08.014

Cortijo S, Charoensawan V, Roudier F, Wigge PA (2018) Chromatin immunoprecipitation sequencing (ChIP-seq) for transcription factors and chromatin factors in *Arabidopsis thaliana* roots: from material collection to data analysis. Methods Mol Biol 1761:231-248. https://doi.org/10.1007/978-1-4939-7747-5_18

Dickinson PJ, Kumar M, Martinho C, Yoo SJ, Lan H, Artavanis G, Charoensawan V, Schöttler MA, Bock R, Jaeger KE, Wigge PA (2018) Chloroplast signaling gates thermotolerance in *Arabidopsis*. Cell Rep 22:1657-1665. https://doi.org/10.1016/j.celrep.2018.01.054

Kim D, Paggi JM, Park C, Bennett C, Salzberg SL (2019) Graph-based genome alignment and genotyping with HISAT2 and HISAT-genotype. Nat Biotechnol 37:907-915. https://doi.org/10.1038/s41587-019-0201-4

Love MI, Huber W, Anders S (2014) Moderated estimation of fold change and dispersion for RNA-seq data with DESeq2. Genome Biol 15:550. https://doi.org/10.1186/s13059-014-0550-8

Martins S, Montiel-Jorda A, Cayrel A, Huguet S, Roux CP-L, Ljung K, Vert G (2017) Brassinosteroid signaling-dependent root responses to prolonged elevated ambient temperature. Nature Commun 8:309. https://doi.org/10.1038/s41467-017-00355-4

Pyl E-T, Piques M, Ivakov A, Schulze W, Ishihara H, Stitt M, Sulpice R (2012) Metabolism and growth in *Arabidopsis* depend on the daytime temperature but are temperature-compensated against cool nights. Plant Cell 24:2443. https://doi.org/10.1105/tpc.112.097188

Zarka DG, Vogel JT, Cook D, Thomashow MF (2003) Cold induction of *Arabidopsis* CBF genes involves multiple ICE (inducer of CBF expression) promoter elements and a cold-regulatory circuit that is desensitized by low temperature. Plant Physiol 133:910-918. https://doi.org/10.1104/pp.103.027169

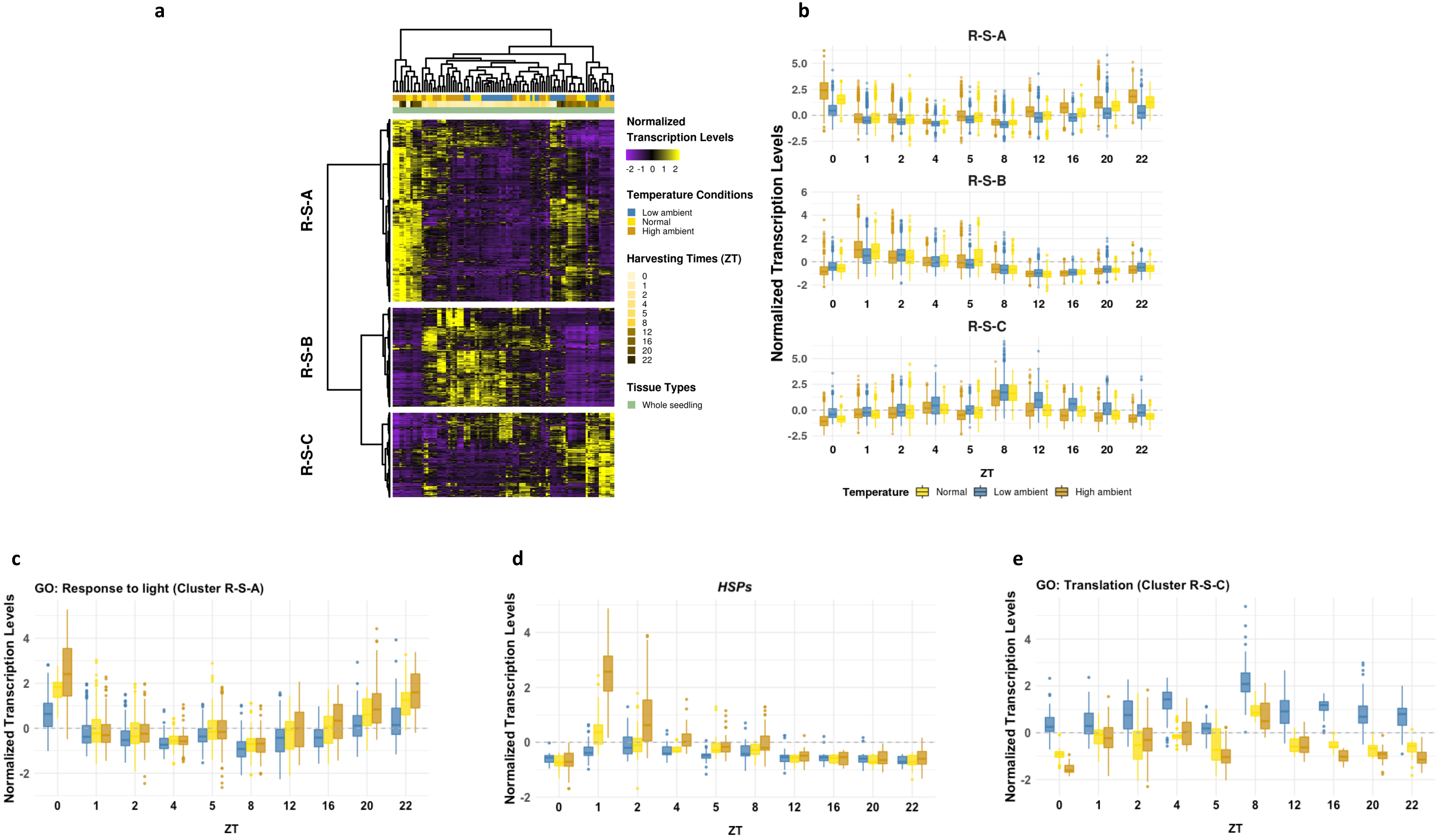
a
R-S-A
R-S-B
R-S-C
Normalized Transcription Levels
-2 -1 0 1 2
Temperature Conditions
Low ambient
Normal
High ambient
Harvesting Times (ZT)
0
1
2
4
5
8
12
16
20
22
Tissue Types
Whole seedling
b
R-S-A
R-S-B
R-S-C
Normalized Transcription Levels
ZT
Temperature
Normal
Low ambient
High ambient
c
GO: Response to light (Cluster R-S-A)
Normalized Transcription Levels
ZT
d
HSPs
Normalized Transcription Levels
ZT
e
GO: Translation (Cluster R-S-C)
Normalized Transcription Levels
ZT

Fig. S9 Temperature-responsive genes from the RNA-seq transcriptomes
a Overall transcription patterns of the temperature HVGs of the integrated RNA-seq transcriptome obtained from the seedling datasets.
b Distributions of the normalized transcription patterns of the seedling HVG clusters across different harvesting times (when the samples were collected, ZT0 – the Zeitgeber time, when the light was on).
c - e Distributions of the normalized transcription levels of: c Forty-six HVGs that belonged to the GO term “response to light” in Cluster R-S-A; d Fourteen HSP genes in Cluster R-S-B; and e Twenty-three HVGs that belonged to the GO term “translation”.
