# Supplementary material for: Large-scale comparative transcriptomic analysis of temperature-responsive genes in Arabidopsis thaliana
Source: Plant Mol Biol. 2022 Jan 1;110(4-5):425–43. doi: 10.1007/s11103-021-01223-y (PMC9646545; doi:10.1007/s11103-021-01223-y)
Supplement: Supplementary file 2 — Supplementary file2 (PDF 3948 kb) [file 11103_2021_1223_MOESM2_ESM.pdf]

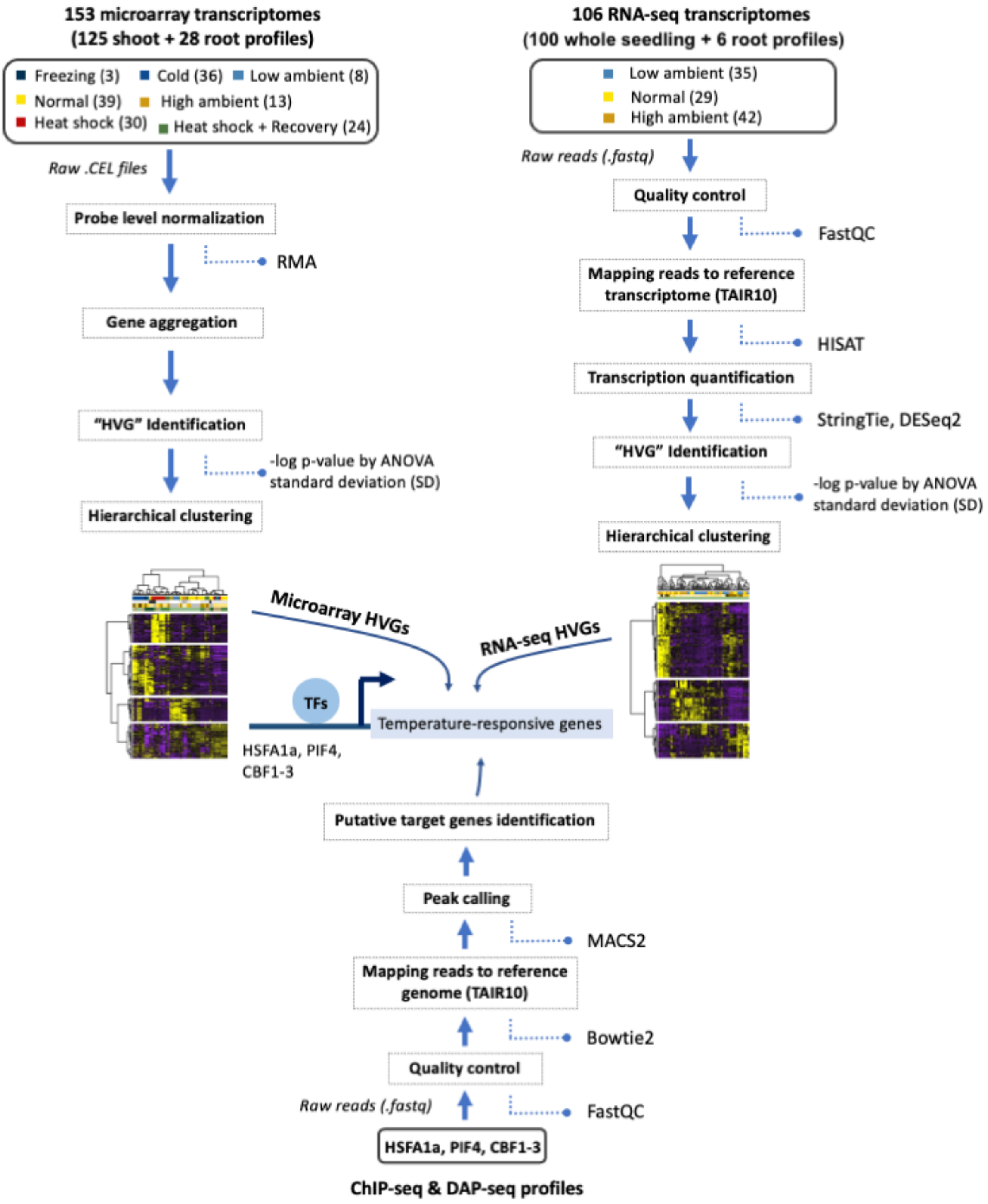

Fig. S1 A scheme of the analysis pipelines for microarray, RNA-seq, ChIP-seq and DAP-seq profiles used in this study

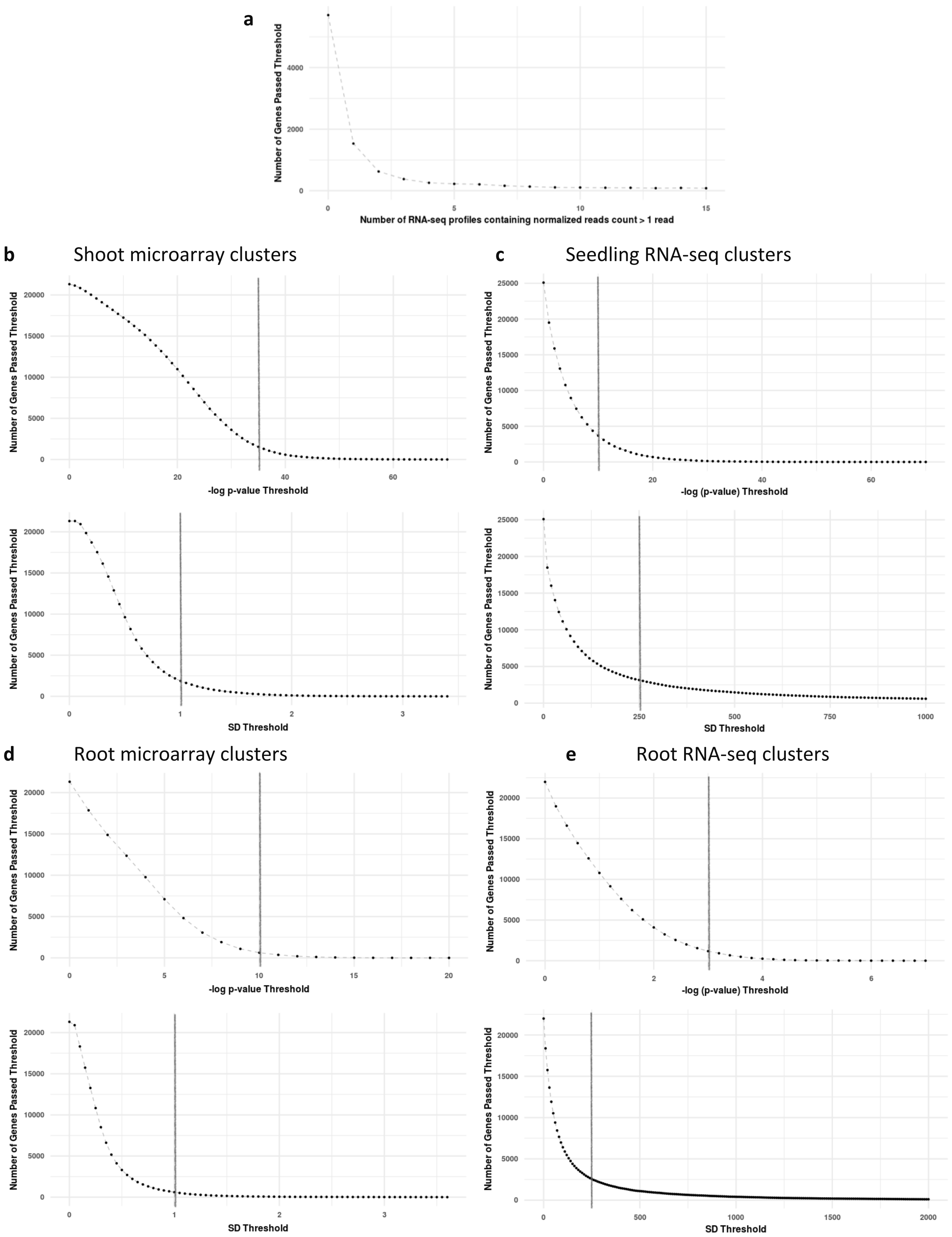

Fig. S2 Identification of highly variable genes (HVGs) from our integrated microarray and RNA-seq transcriptomic profiles

a Numbers of RNA-seq profiles containing normalized read count lower than 1.

b - e Numbers of genes passing the cut-off thresholds of p-values and SDs in: **b** Integrated shoot microarray transcriptomes (M-Ss, cutoffs are -log p-value > 35 and SD > 1), **c** Integrated RNA-seq shoot transcriptomes (R-Ss, -log p-value > 10 and SD > 250), **d** Integrated microarray root transcriptomes (M-Rs, -log p-value > 10 and SD > 1), and **e** Integrated RNA-seq root transcriptomes (R-Rs, -log p-value > 3 and SD > 250)

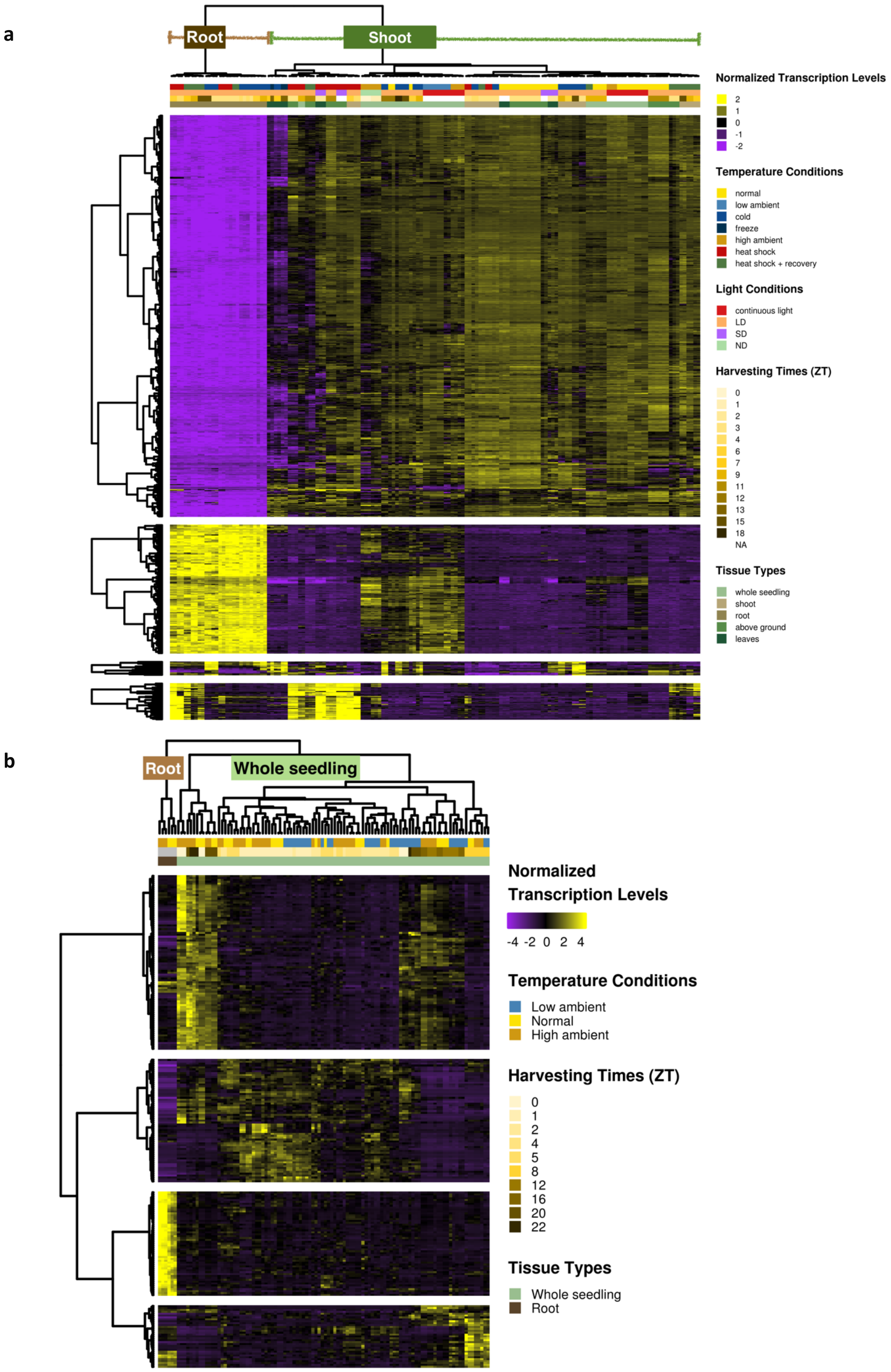

Fig. S3 Clusters of the complete sets of integrated transcriptomic profiles in this study (from all the tissue types) was separated by tissue types

a Microarray datasets (labeled in brown and green for the root and the shoot, respectively)

b RNA-seq datasets (labeled in brown and green for the root and the seedling, respectively)

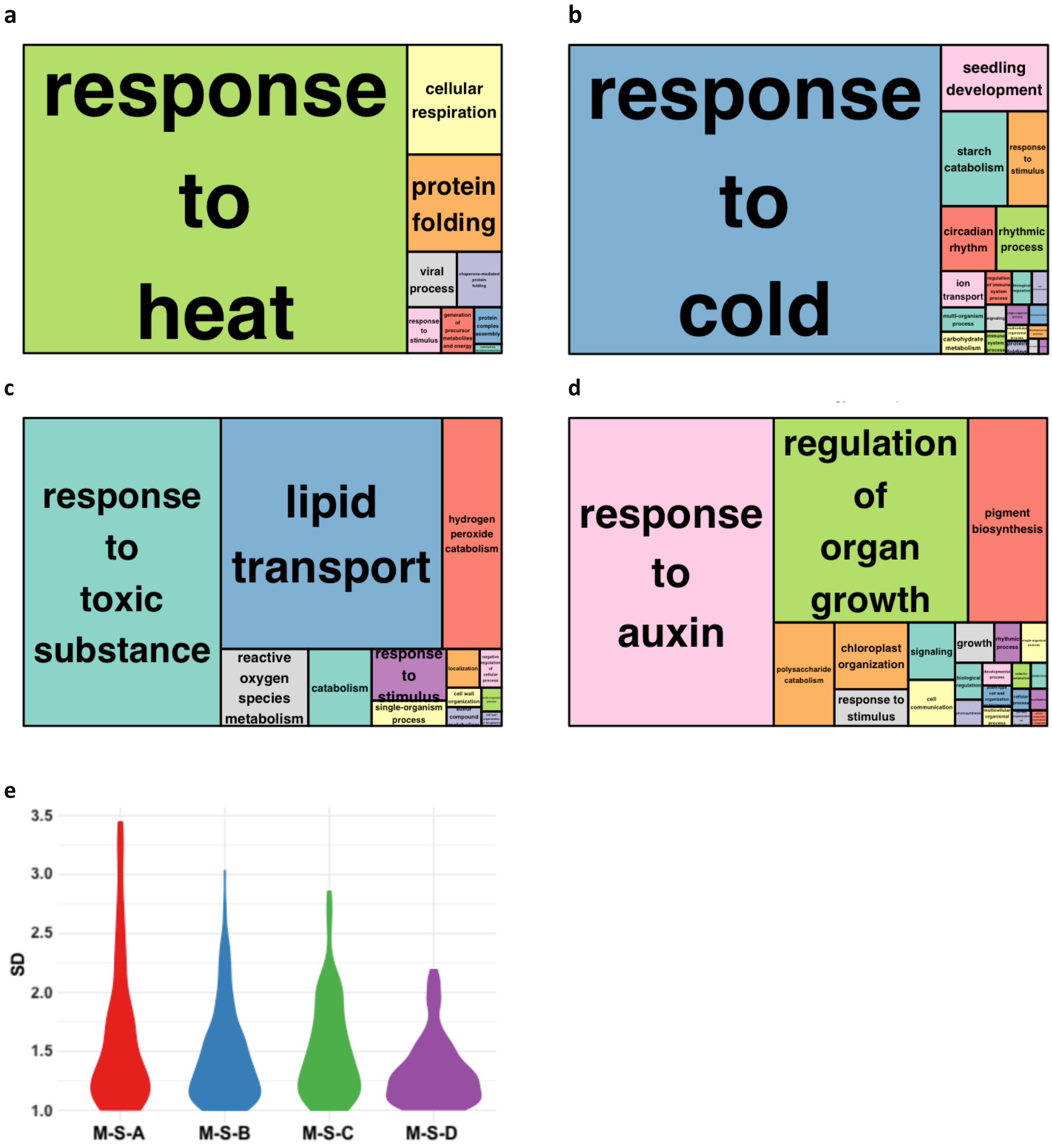

Fig. S4 Enriched functions of HVGs from Microarray-Shoot (M-S) clusters
a - d Non-redundant GO terms enrichment analysis of microarray HVGs in:
a Cluster M-S-A, **b** Cluster M-S-B, **c** Cluster M-S-C and **d** Cluster M-S-D
e SD distributions of the HVGs in the M-S clusters

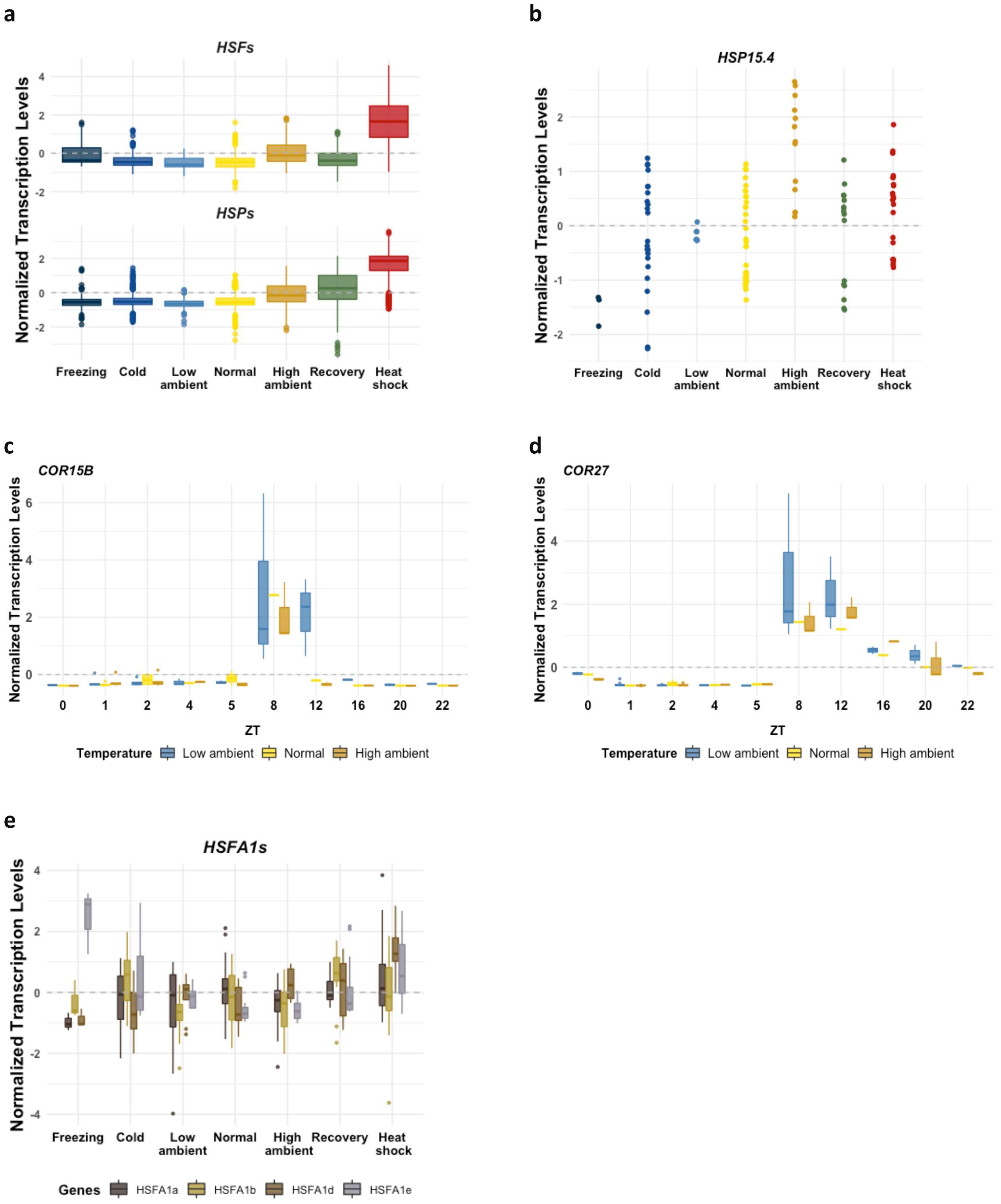

Fig. S5 Normalized transcription levels of selected HVGs from microarray and RNA-seq temperature transcriptomes

a – e Distributions of the normalized transcription levels under different temperature conditions of:

a seven *HSF* (*HSFA2*, *HSFB1*, *HSFB2A*, *HSFB2B*, *HSFA3*, *HSFA7A*, and *HSFA7B*) and 26 *HSP* genes in Cluster M-S-A, **b** *HSP15.4* in Cluster M-S-C, **c** *COR15B* and **d** *COR27* in Cluster R-S-C, and **e** *HSFA1s* (including *HSFA1a*, *HSFA1b*, *HSFA1d* and *HSFA1e*)

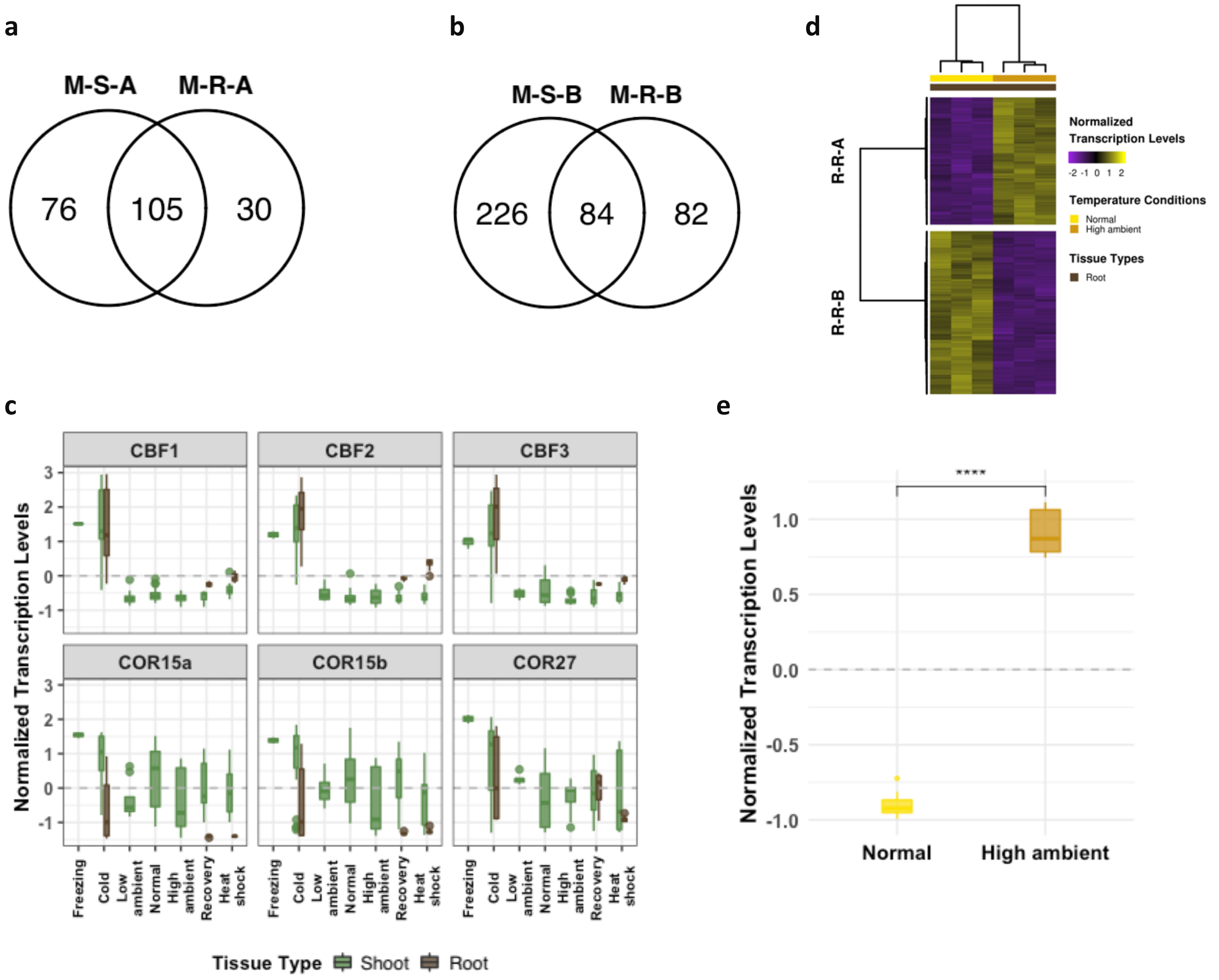

Fig. S6 Temperature-responsive genes from temperature transcriptomes of plant's root parts
a - b Overlapping HVGs between **a** Clusters M-S-A and Cluster M-R-A and **b** Clusters M-S-B and M-R-B
c Distributions of the normalized transcription levels of *CBF1, CBF2, CBF3, COR15a-b* and *COR27* in the shoots and roots under different temperature-specific conditions. In this case, the transcription levels from the shoots and roots were re-normalized together in order to compare relative transcription between the tissues
d Overall transcription patterns of the temperature HVGs of the RNA-seq root transcriptome obtained from the study by Martins and colleagues (Martins et al. 2017)
e Normalized transcription levels of six *HSP* genes (*HSP18.5*, *HSP23.5*, *HSP70-2*, *HSP70-4*, *HSP90-1* and *CLPB1*) in Cluster R-R-A under normal and high ambient temperature. Wilcoxon test was performed to statistically assess the differences between the normal and high ambient temperature condition, *, **, ***, **** indicate p-values of ≤ 0.05, ≤ 0.01, ≤ 0.001, ≤ 0.0001, respectively. ns = not significance

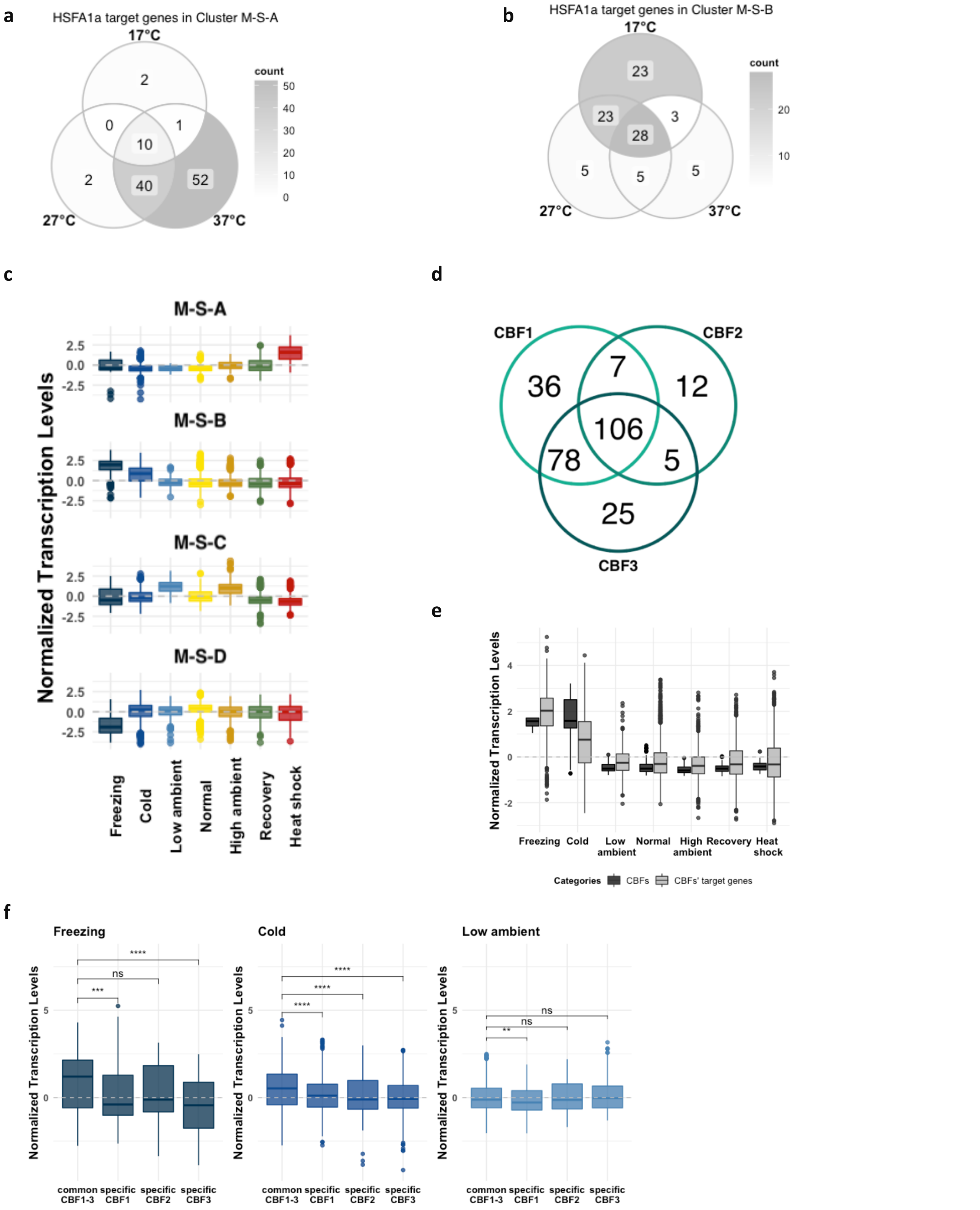

Fig. S7 Characteristics of genome-wide binding occupancy of HSFA1a (Cortijo et al. 2017), PIF4 (Oh et al. 2012) and CBFs (O'Malley et al. 2016).

a - b Numbers of HSFA1a's target genes identified in **a** Cluster M-S-A and **b** Cluster M-S-B

c Normalized transcription levels of the PIF4 target genes based on the shoot microarray transcriptomes

d Numbers of the identified target genes of CBFs

e Normalized transcription levels of *CBFs* and their direct target genes across different temperature conditions based on the shoot microarray transcriptomes

f Normalized transcription levels of the CBF target genes in the low temperature conditions, namely freezing, cold, and low ambient temperature

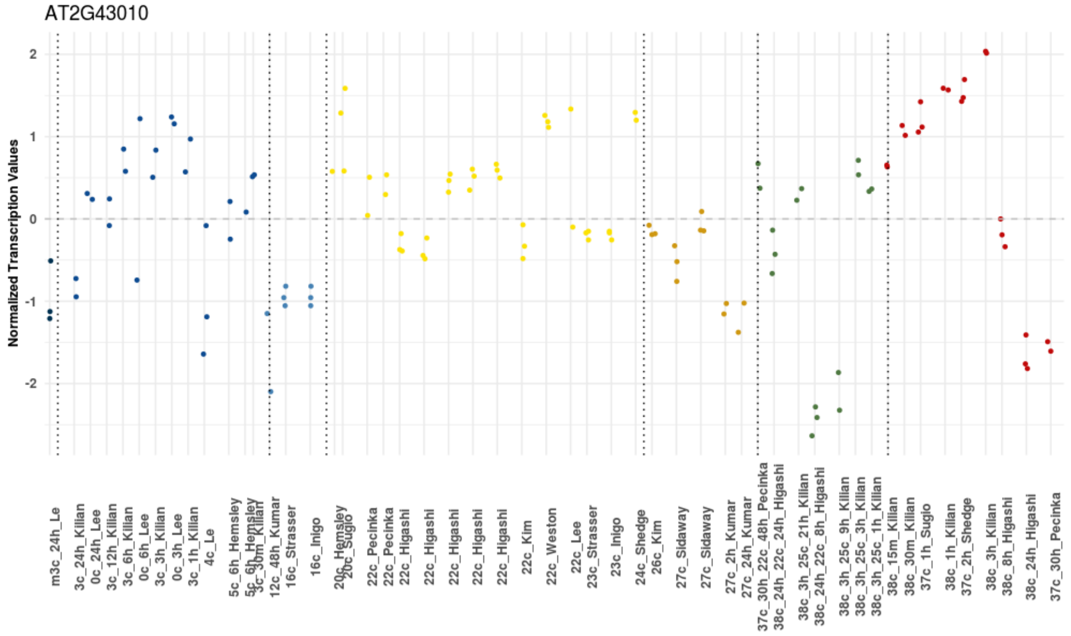

Fig. S8 Normalized transcription levels from shoot microarray transcriptomes of *PIF4 (AT2G43010)*

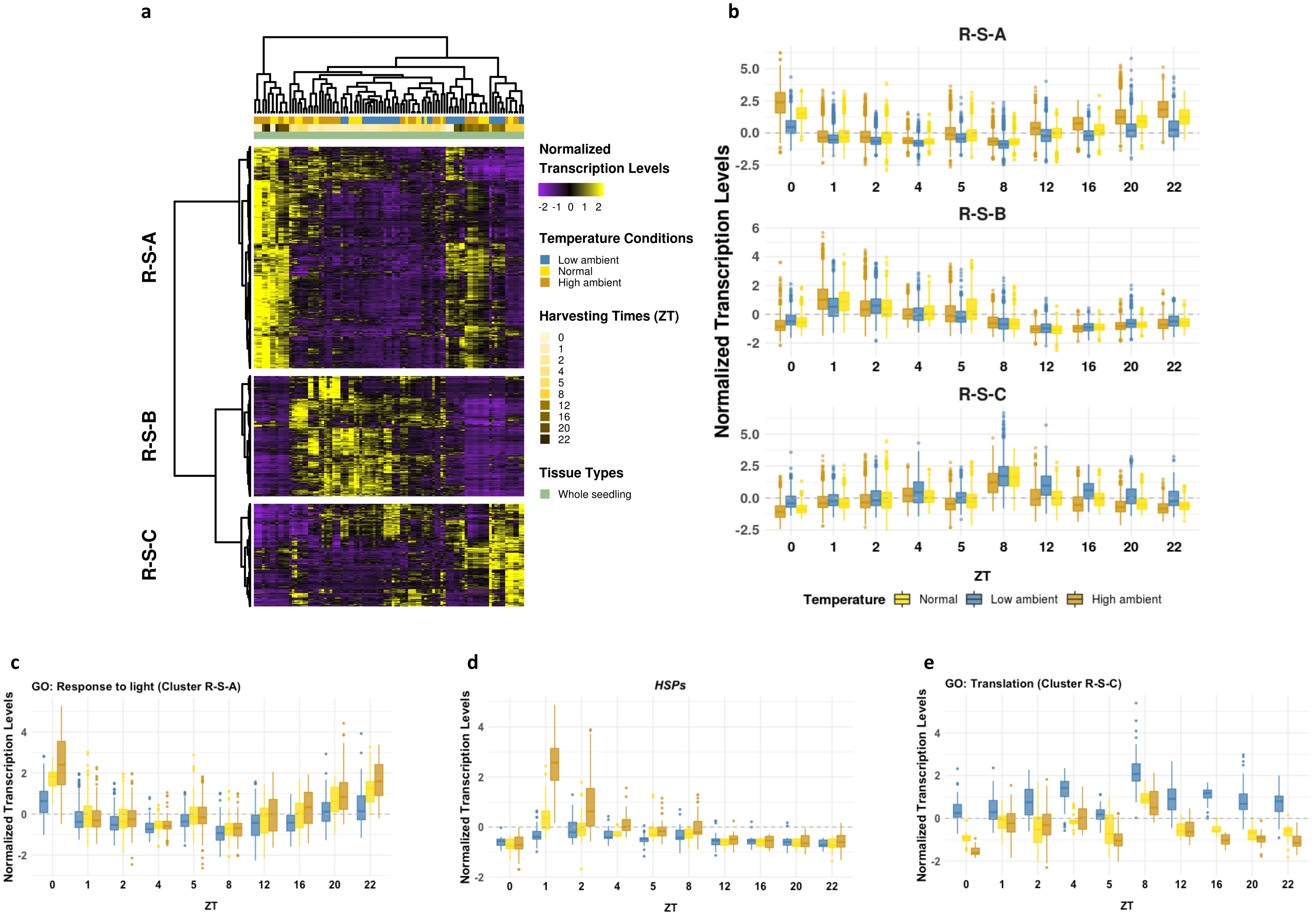
a
b
c
d
e
R-S-A
R-S-B
R-S-C
Normalized Transcription Levels
-2 -1 0 1 2
Temperature Conditions
Low ambient
Normal
High ambient
Harvesting Times (ZT)
0
1
2
4
5
8
12
16
20
22
Tissue Types
Whole seedling
Normalized Transcription Levels
ZT
Temperature
Normal
Low ambient
High ambient
GO: Response to light (Cluster R-S-A)
HSPs
GO: Translation (Cluster R-S-C)

Fig. S9 Temperature-responsive genes from the RNA-seq transcriptomes

a Overall transcription patterns of the temperature HVGs of the integrated RNA-seq transcriptome obtained from the seedling datasets.

b Distributions of the normalized transcription patterns of the seedling HVG clusters across different harvesting times (when the samples were collected, ZT0 – the Zeitgeber time, when the light was on).

c - e Distributions of the normalized transcription levels of: c Forty-six HVGs that belonged to the GO term “response to light” in Cluster R-S-A; d Fourteen HSP genes in Cluster R-S-B; and e Twenty-three HVGs that belonged to the GO term “translation”

Temperature transcriptomic profiles analyzed in this study

Microarray

1. Hemsley PA et al. (2014) The *Arabidopsis* Mediator Complex Subunits MED16, MED14, and MED2 Regulate Mediator and RNA Polymerase II Recruitment to CBF-Responsive Cold-Regulated Genes. Plant Cell 26:465. https://10.1105/tpc.113.117796
2. Higashi Y, Okazaki Y, Myouga F, Shinozaki K, Saito K (2015) Landscape of the lipidome and transcriptome under heat stress in *Arabidopsis thaliana*. Sci Rep 5:10533. https://10.1038/srep10533
3. Iñigo S, Alvarez Mariano J, Strasser B, Califano A, Cerdán Pablo D (2012) PFT1, the MED25 subunit of the plant Mediator complex, promotes flowering through CONSTANS dependent and independent mechanisms in Arabidopsis. Plant J 69:601-612. https://10.1111/j.1365-313X.2011.04815.x
4. Kilian J et al. (2007) The AtGenExpress global stress expression data set: protocols, evaluation and model data analysis of UV-B light, drought and cold stress responses. Plant J 50:347-363. https://10.1111/j.1365-313X.2007.03052.x
5. Kim BB PK, Grant JS, Hicks JS, Zellous DC, Anderson DR (2016) Shade-Inducible Gene Expression Change in *Arabidopsis thaliana* at Different Temperatures. Am J Plant Sci 7:352-423. https://10.4236/ajps.2016.72035
6. Kumar SV, Wigge PA (2009) H2A.Z-Containing Nucleosomes Mediate the Thermosensory Response in *Arabidopsis*. Cell 140:136-147. https://doi.org/10.1016/j.cell.2009.11.006
7. Le MQ, Pagter M, Hincha DK (2015) Global changes in gene expression, assayed by microarray hybridization and quantitative RT-PCR, during acclimation of three *Arabidopsis thaliana* accessions to sub-zero temperatures after cold acclimation. Plant Mol Biol 87:1-15. https://10.1007/s11103-014-0256-z
8. Lee BH, Henderson DA, Zhu JK (2005) The *Arabidopsis* cold-responsive transcriptome and its regulation by ICE1. Plant Cell 17:3155-3175. https://10.1105/tpc.105.035568
9. Pecinka A, Dinh HQ, Baubec T, Rosa M, Lettner N, Scheid OM (2010) Epigenetic Regulation of Repetitive Elements Is Attenuated by Prolonged Heat Stress in *Arabidopsis*. Plant Cell 22:3118. https://10.1105/tpc.110.078493
10. Shedge V, Davila J, Arrieta-Montiel MP, Mohammed S, Mackenzie SA (2010) Extensive Rearrangement of the *Arabidopsis* Mitochondrial Genome Elicits Cellular Conditions for Thermotolerance. Plant Physiol 152:1960. https://10.1104/pp.109.152827
11. Sidaway-Lee K, Costa MJ, Rand DA, Finkenstadt B, Penfield S (2014) Direct measurement of transcription rates reveals multiple mechanisms for configuration of the *Arabidopsis* ambient temperature response. Genome Biol 15:R45. https://doi.org/10.1186/gb-2014-15-3-r45
12. Strasser B, Alvarez MJ, Califano A, Cerdan PD (2009) A complementary role for ELF3 and TFL1 in the regulation of flowering time by ambient temperature. Plant J 58:629-640. https://10.1111/j.1365-313X.2009.03811.x
13. Sugio A, Dreos R, Aparicio F, Maule AJ (2009) The cytosolic protein response as a subcomponent of the wider heat shock response in *Arabidopsis*. Plant Cell. https://doi.org/10.1105/tpc.108.062596
14. Weston DJ, Karve AA, Gunter LE, Jawdy SS, Yang X, Allen SM, Wullschleger SD (2011) Comparative physiology and transcriptional networks underlying the heat shock response in *Populus trichocarpa*, *Arabidopsis thaliana* and *Glycine max*. Plant, cell & environment 34:1488-1506. https://10.1111/j.1365-3040.2011.02347.x

RNA-seq

1. Cortijo S et al. (2017) Transcriptional Regulation of the Ambient Temperature Response by H2A.Z Nucleosomes and HSF1 Transcription Factors in *Arabidopsis*. Mol Plant 10:1258-1273. https://doi.org/10.1016/j.molp.2017.08.014
2. Dickinson PJ et al. (2018) Chloroplast Signaling Gates Thermotolerance in *Arabidopsis*. Cell Rep 22:1657-1665. https://doi.org/10.1016/j.celrep.2018.01.054
3. Duruflé H et al. (2017) Cell wall modifications of two *Arabidopsis thaliana* ecotypes, *Col* and *Sha*, in response to sub-optimal growth conditions: An integrative study. Plant Sci 263:183-193. https://10.1016/j.plantsci.2017.07.015
4. Ezer D et al. (2017a) The evening complex coordinates environmental and endogenous signals in *Arabidopsis*. Nat Plants 3:17087. https://10.1038/nplants.2017.87

5. Ezer D et al. (2017b) The G-Box Transcriptional Regulatory Code in *Arabidopsis*. Plant Physiol 175:628-640. https://10.1104/pp.17.01086
6. Martins S, Montiel-Jorda A, Cayrel A, Huguet S, Roux CP-L, Ljung K, Vert G (2017) Brassinosteroid signaling-dependent root responses to prolonged elevated ambient temperature. Nature Commun 8:309. https://doi.org/10.1038/s41467-017-00355-4
7. Tasset C et al. (2018) POWERDRESS-mediated histone deacetylation is essential for thermomorphogenesis in *Arabidopsis thaliana*. PLoS Genet 14:e1007280. https://10.1371/journal.pgen.1007280
8. Zhu W et al. (2015) Natural Variation Identifies ICARUS1, a Universal Gene Required for Cell Proliferation and Growth at High Temperatures in *Arabidopsis thaliana*. PLoS Genet 11:e1005085. https://10.1371/journal.pgen.1005085

DNA-binding profiles analyzed in this study

ChIP-seq

1. Cortijo S et al. (2017) Transcriptional Regulation of the Ambient Temperature Response by H2A.Z Nucleosomes and HSF1 Transcription Factors in *Arabidopsis*. Mol Plant 10:1258-1273. https://doi.org/10.1016/j.molp.2017.08.014
2. Oh E, Zhu J-Y, Wang Z-Y (2012) Interaction between BZR1 and PIF4 integrates brassinosteroid and environmental responses. Nat Cell Biol 14:802-809. https://doi.org/10.1038/ncb2545

DAP-seq

1. O'Malley RC et al. (2016) Cistrome and Epicistrome Features Shape the Regulatory DNA Landscape. Cell 165:1280-1292. https://doi.org/10.1016/j.cell.2016.04.038
